# Chemical Constructions, Mechanism and Biological Interpretation of Nitroreductase-Responsive Systems: From Hypoxia-Associated Imaging to Activatable Therapy

**DOI:** 10.3390/molecules31193465

**Published:** 2026-09-29

**Authors:** Xinye Lin, Junhan Yang, Jiawei Huang, Ping Zhang, Xinwei Yang, Daliang Li

**Affiliations:** 1 College of Life Sciences, Fujian Normal University, Fuzhou 350117, China; 2 Fujian Key Laboratory of Innate Immune Biology, Biomedical Research Center of South China, College of Life Sciences, Fujian Normal University, Fuzhou 350117, China

**Keywords:** nitroreductase-responsive systems, nitroaromatic reduction, reaction architecture, operational nitro-reducing activity, hypoxia-associated imaging, activatable fluorescence, logic-gated activation, biological validation

## Abstract

Nitroreductase (NTR)-responsive systems convert the enzymatic reduction of nitroaromatic substrates into detectable signals, molecular retention, supramolecular assembly, or therapeutic activation. Their interpretation is nevertheless complicated by the distinct catalytic properties of bacterial NTRs, experimentally introduced or engineered NTR systems, and endogenous mammalian nitro-reducing enzymes. In particular, an NTR-responsive signal in mammalian tissues should not automatically be interpreted as a direct measurement of oxygen concentration or as evidence for the activity of a single endogenous “NTR.” This review analyzes NTR-responsive systems through an integrated framework that connects catalytic source, chemical event, signal-transduction mechanism, and biological application. We first distinguish defined bacterial NTRs, engineered or experimentally introduced NTR systems, and endogenous mammalian nitro-reducing activity. We then describe the recognition, linker, and output modules used in molecular design and compare three major reaction architectures: direct reduction, reduction-triggered cleavage, and reduction-triggered retention or assembly. Photoinduced electron transfer (PET), intramolecular charge transfer (ICT), fluorescence resonance energy transfer (FRET), aggregation-induced emission (AIE), chemiluminescence, photoacoustic imaging, and fluorine nuclear magnetic resonance (^19^F NMR) are discussed as signal-transduction modalities rather than as independent activation mechanisms. Multi-input sensing, activatable therapy, and the validation of these systems in complex biological models are subsequently evaluated. Particular emphasis is placed on product-level confirmation, enzyme attribution, oxygen-dependent interpretation, calibration, pharmacokinetics, biodistribution, and safety. By linking chemical activation to the biochemical source of reduction and to the limitations of biological inference, this review provides criteria for selecting, validating, and translating NTR-responsive systems for hypoxia-associated imaging and activatable therapy.

## 1. Introduction

Hypoxia is a common feature of many solid tumors and is associated with altered metabolism, treatment resistance, and spatial heterogeneity. Nitroaromatic reduction has therefore been widely used to report reductive microenvironments. In bacteria, NTRs are flavin-dependent enzymes with defined catalytic identities. In mammalian cells, however, activation of a nitroreductase (NTR)-responsive probe is usually produced by a substrate-dependent pool of reductases whose contributions vary with oxygen tension, cofactor availability, probe structure, and subcellular localization. An NTR-responsive signal should consequently be interpreted first as evidence of probe reduction under the stated conditions, not automatically as a measurement of a unique endogenous enzyme or of oxygen concentration itself.

The field has expanded along three linked axes: from simple intensity turn-on to ratiometric and multimodal outputs; from single-input activation to targeting and logic-gated recognition; and from imaging to conditional payload release or phototherapy. These developments could improve contrast, retention, or functional scope, but they also add determinants of signal such as delivery, linker fragmentation, assembly, target abundance, and treatment-induced changes in the microenvironment. A useful comparison must therefore separate the chemical reaction architecture from the optical transduction mechanism and from the biological application. Furthermore, for each representative system, the relevant questions are whether the reaction product was confirmed, which enzyme source and cofactor conditions were used, whether competing reductases or single-input controls were tested, how the output was calibrated, and what additional variables were introduced by targeting, retention, or therapy. The term “system” encompasses small-molecule reporters, self-immolative conjugates, and nanoformulations in which nitroreduction is an explicit activation step. Native bacterial diagnostics and engineered NTR–prodrug or ablation models are retained as separate biological contexts; they are not treated as directly comparable measures of endogenous mammalian hypoxia. Platforms responsive only to pH, glutathione (GSH), viscosity, or other tumor-associated variables are excluded from the principal comparison.

To address potential interference, chemical selectivity and biological specificity must be distinguished when evaluating a new sensor. A probe may undergo the intended nitro-to-amine conversion in a purified-enzyme assay and still be activated by several reductases in cells, whereas a highly localized in vivo signal may arise from preferential delivery rather than selective catalysis. This distinction is especially important for mammalian studies, where the term NTR often compresses a heterogeneous set of flavin-dependent and other reductive activities into a single operational label [1,2,3,4,5]. Throughout this review, selectivity is therefore discussed at the level actually demonstrated: reaction selectivity, enzyme-source selectivity, cell-state association, or lesion-level contrast.

Cross-study comparison is also constrained by nonstandard experimental conditions. Reported response times and detection limits vary with enzyme source and concentration, nicotinamide adenine dinucleotide (NADH) or nicotinamide adenine dinucleotide phosphate (NADPH) level, cosolvent fraction, oxygenation, temperature, incubation sequence, and the method used to calculate the limit of detection. Rather than ranking probes from isolated numerical values, the discussion relates each metric to its assay context and to the validation that accompanies it. This approach retains the breadth of the literature while avoiding the false precision created by tables that place non-equivalent measurements side by side.

Table 1 provides a direct comparison with recent reviews. The present article differs by separating defined bacterial NTRs, engineered NTR systems, and endogenous mammalian nitro-reducing activity as distinct biological settings; treating direct reduction, fragmentation, and retention or assembly as reaction architectures rather than organizing the literature by fluorophore or disease alone; placing PET, ICT, FRET, AIE, photoacoustic, chemiluminescent, and ^19^F NMR readouts at the signal-transduction level; comparing imaging and therapy within one chemical-to-biological framework; and emphasizing enzyme attribution, product confirmation, calibration, pharmacokinetics, and safety.

Several recent reviews have examined NTR-responsive probes and related hypoxia-associated imaging or therapeutic systems from complementary perspectives [1,2,6,7], including the reviews by Shang et al. in 2024, Verma et al. in 2024, Tran et al. in 2025, and Ji et al. in 2026. These publications collectively demonstrate the rapid development of NTR-responsive molecular platforms and provide valuable summaries of representative fluorophores, sensing mechanisms, biological applications, or therapeutic strategies. Their appearance also makes it necessary to define explicitly the analytical scope and added value of the present review.

The present review does not classify systems solely according to fluorophore type, emission wavelength, imaging modality, or disease application. Instead, it uses a sequential framework. The review proceeds from catalytic basis to modular probe design, dominant reaction architectures, and application-level control. Section 2 distinguishes bacterial NTRs, endogenous mammalian nitro-reducing activity, and engineered NTR systems. Section 3 defines recognition, linker, and output modules. Section 4 compares direct reduction, reduction-triggered cleavage, and reduction-triggered retention or assembly while treating photoinduced electron transfer (PET), intramolecular charge transfer (ICT), fluorescence resonance energy transfer (FRET), aggregation-induced emission (AIE), chemiluminescence, photoacoustic imaging, and fluorine nuclear magnetic resonance (^19^F NMR) as transduction modalities. Section 5 examines multi-input activation, activatable therapy, and the validation required in complex models.

The conceptual advance of the present review is therefore not another fluorophore- or application-based classification. Instead, it separates catalytic source, chemical reaction architecture, signal transduction, and biological interpretation as distinct analytical dimensions. This framework prevents NTR-responsive signals in mammalian systems from being conflated with a unique endogenous enzyme or with direct oxygen measurement, while enabling comparison of validation requirements across imaging and therapeutic platforms.

The central argument is that molecular complexity is justified only when it resolves a defined analytical, delivery, or therapeutic limitation. Ratiometric outputs may reduce concentration-dependent artifacts, logic gates may suppress specified background pathways, and self-immolative or retention reactions may improve payload release or localization. Conversely, each added component can slow kinetics, obscure the relationship between signal and initial reductase activity, or introduce new pharmacokinetic and safety liabilities. Artificial intelligence/computer-aided drug design (AI/CADD) and near-infrared-II (NIR-II) methods are therefore considered prospective tools rather than established routes to clinical implementation (Figure 1).

### Review Scope and Comparison Strategy

This article is a critical narrative review rather than a systematic meta-analysis. The literature is used to define mechanistic classes and evidence boundaries, not to estimate the prevalence of any architecture or to rank non-equivalent assays. The principal comparison includes systems in which nitroreduction is an explicit activation step; platforms responsive only to pH, GSH, viscosity, or other microenvironmental variables are excluded from the main analysis.

Representative investigations are compared along three orthogonal axes: the chemical event after nitroreduction, the signal-transduction mode, and the biological application. Original studies are used to illustrate distinct architectures, validation strategies, and translational boundaries. Native bacterial detection and engineered NTR-prodrug systems are retained as separate contexts and are not treated as direct measures of endogenous mammalian hypoxia.

## 2. Fundamental Characteristics and Biological Context of Nitroreductase-Responsive Reduction

In this review, the term “NTR” is used only when referring to a defined nitroreductase enzyme, a characterized bacterial NTR family, or an experimentally introduced NTR system. When the responsible mammalian enzyme has not been established, the terms “nitro-reducing activity” or “NTR-responsive signal” are used instead. This distinction is necessary because bacterial NTRs, mammalian NQO1, cytochrome P450 reductase, and other flavoprotein reductases differ in substrate preference, cofactor dependence, oxygen sensitivity, subcellular distribution, and physiological function.

### 2.1. Enzyme Classification, Structure, and Catalytic Mechanism

Bacterial nitroreductases are flavin-dependent oxidoreductases that reduce nitroaromatic substrates using NADH- or NADPH-derived reducing equivalents. Their established roles include xenobiotic metabolism, bioremediation, and enzyme-prodrug systems. In mammalian imaging studies, however, “NTR” is often used operationally for substrate-dependent nitro-reducing activity and should not imply a single conserved oxygen-insensitive nitroreductase (NfsB)-like protein [1,2].

Bacterial NTRs are commonly grouped by oxygen sensitivity (Figure 2). Type I enzymes are oxygen-insensitive two-electron reductases; Escherichia coli NfsA and NfsB belong to this class. Type II activity proceeds through one-electron transfer to form a nitro radical anion that is readily reoxidized by oxygen, so productive reduction is favored under low-oxygen conditions [3,4].

This classification describes catalytic pathways, not tumor expression. Type I bacterial NTRs can reduce suitable substrates under both aerobic and anaerobic conditions [4]. Mammalian tumor imaging instead detects the combined activity of endogenous flavoproteins and other reductases toward a particular probe; the responsible enzyme population can vary among cell types and substrates [1,2].

The classical Type I/Type II distinction is useful for describing electron-transfer chemistry, but it does not define a universal family of mammalian hypoxia enzymes. Even among bacterial nitroreductases, sequence diversity, active-site geometry, flavin environment, and cofactor preference produce substantial differences in substrate range and oxygen dependence [3,4,8,9]. NfsA, NfsB, and related enzymes should therefore be named explicitly when they are used, and results obtained with a commercial preparation should not be generalized without information on its source and composition.

Kinetic interpretation requires more than an endpoint fluorescence curve. Substrate turnover can be limited by binding, flavin reduction, electron transfer to the nitro group, oxygen-mediated back oxidation, or product release. Recent structural and kinetic work on bacterial nitroreductases reinforces the breadth of this superfamily and the need to report catalytic context [1,8,10]. For probe studies, a minimal description should include enzyme identity, concentration, cofactor type and concentration, oxygen condition, and evidence for the expected product. These variables are not ancillary methods details; they determine what the optical response means.

Many bacterial NTRs are homodimeric flavoproteins with an FMN-binding region and a binding environment for NADH or NADPH. The reduced nicotinamide cofactor transfers reducing equivalents to FMN, and reduced FMN subsequently transfers electrons to the nitroaromatic substrate. Substrate scope and oxygen sensitivity depend on enzyme family and active-site architecture; these properties should not be generalized from one bacterial enzyme to all cellular reductases.

Crystal structures of Escherichia coli NfsB show a dimeric FMN-dependent enzyme with an active site formed at the subunit interface. The flavin and surrounding residues organize substrate binding and electron transfer, while active-site plasticity contributes to broad nitroaromatic substrate tolerance [9]. These structural features should be described specifically for NfsB rather than generalized to all NTR-like reductases.

The general activation chemistry of NTR-responsive probes involves reduction of an aromatic nitro group through nitroso and hydroxylamine intermediates to the corresponding amine (Figure 3). For two-electron reductases, NAD(P)H first reduces the flavin cofactor, after which reduced flavin supplies successive reducing equivalents to the substrate. One-electron pathways generate nitro radical anions that can be reoxidized by molecular oxygen, explaining their oxygen sensitivity. This chemistry underlies common designs in which reduction changes electron distribution or initiates linker cleavage.

### 2.2. Biological Interpretation in Mammalian Hypoxia and Engineered NTR Systems

#### 2.2.1. Endogenous Mammalian Nitro-Reducing Activity

Solid-tumor hypoxia is associated with aggressive behavior and resistance to radiotherapy and chemotherapy. Hypoxic mammalian tissues can exhibit substrate-dependent nitro-reducing activity from multiple oxidoreductases. This operational activity can support conditionally activated probe responses, but it does not establish increased expression of a single endogenous NTR or of any one reductase (Figure 4) [4]. NTR-responsive imaging uses reduction-dependent activation as a functional proxy for a reductive, often hypoxic microenvironment rather than direct proof of a single enzyme [2]. Py-SiRh-NTR (Figure 5) and fol-BODIPY (Figure 6) demonstrated oxygen-dependent signal changes in cells and tumor-bearing mice [11,12], while ratiometric probes extended this approach to three-dimensional spheroids [13]. These studies support hypoxia-associated imaging, but their outputs remain functions of probe delivery, enzyme composition, cofactor availability, and optical attenuation.

Temporal integration is another important consideration in bioimaging. Irreversible probes can retain the chemical history of delivery and reduction after oxygenation has changed, whereas reversible or rapidly cleared systems may report a narrower time window. This distinction is relevant when imaging treatment response, intermittent hypoxia, or organelle-specific stress. Time-course experiments should therefore be interpreted together with probe retention and product stability, not only with the nominal oxygen condition applied at the imaging endpoint.

Endogenous mammalian nitroreduction arises from a substrate-governed mixture of oxidoreductases rather than a single NfsA/NfsB-homologous enzyme. NAD(P)H:quinone oxidoreductase 1 (NQO1) can mediate the two-electron reduction of suitable nitroaromatic substrates [14,15,16]; NADPH-cytochrome P450 oxidoreductase and other flavoprotein reductases can contribute in a substrate- and context-dependent manner. Oxygen-associated readouts arise from reaction-level redox competition: one-electron reduction generates O2-susceptible nitro radical anions that can undergo futile cycling and reactive oxygen species (ROS) production, whereas two-electron reduction can bypass radical intermediates. Low oxygen can potentiate net reductive turnover without implying hypoxia-specific enzyme upregulation. Definitive reductase assignment requires convergent evidence because recombinant activity, pharmacological blockade, genetic manipulation, and spectrometric product quantification each have limitations. These caveats also apply to inflammatory and infectious models, where multiple biological sources can confound simple enzyme-centric interpretation.

#### 2.2.2. Native Microbial and Engineered NTR Concerns

Compared with endogenous mammalian imaging, the bacterial NTR/CB1954 system represents a distinct setting in which the activating enzyme is deliberately introduced and its identity is defined. Expression of bacterial NTR markedly sensitized tumor cells to CB1954 and could generate a bystander effect through diffusible cytotoxic metabolites [5,17,18]. Early clinical investigation of adenoviral NTR delivery documented local transgene expression and biological activity but also exposed limitations in distribution, dose, and toxicity [19]. These studies validate an engineered enzyme–prodrug switch; they do not establish that endogenous mammalian imaging probes measure an equivalent enzyme system.

Different biological settings therefore require distinct interpretive criteria. Endogenous mammalian imaging asks whether a nitroaromatic substrate is reduced in a disease-associated microenvironment; bacterial detection asks whether microbial metabolism produces sufficient activation for diagnosis; and engineered NTR systems ask whether an introduced enzyme can control ablation or prodrug conversion. Although the basic chemistry may be related, enzyme identity, background sources, and validation standards differ. Combining these settings in a single claim of NTR specificity obscures rather than strengthens the scope of the field [17,18,20,21,22,23,24].

#### 2.2.3. Evidence Boundaries Across Biological Settings

Taken together, imaging and engineered-enzyme studies support nitro-reducing chemistry as a useful functional input [1,2,5,13,17,18,19,25], but defensible biological claims depend on the model (Table 2). Purified bacterial enzyme assays can establish substrate turnover and cofactor dependence; mammalian-cell experiments generally establish probe reduction under specified oxygen and metabolic conditions; and animal imaging can demonstrate lesion-associated contrast and distribution. Attribution to one endogenous reductase, quantitative tissue oxygenation, or human diagnostic utility requires independent molecular or physiological measurements that are rarely included in the current literature.

## 3. Modular Design Principles of NTR-Responsive Molecular Systems

### 3.1. Recognition Units and Substrate Compatibility

Nitrobenzene, nitrobenzyl, nitroimidazole, and nitro groups directly conjugated to reporters are not interchangeable recognition units. Their reduction potentials, steric accessibility, enzyme-family compatibility, oxygen sensitivity, and aqueous stability determine both apparent kinetics and the reductase population capable of activation [1,3,4,7,8]. Trigger selection should therefore begin with the intended chemical endpoint—direct spectral reconfiguration, fragmentation, or retention—rather than the generic label “NTR-responsive” (Figure 7).

Regiochemistry and substitution define how reduction is translated into output. Para- and ortho-nitrobenzyl arrangements can support self-immolative relays after amine formation, whereas meta substitution more often favors electronic modulation. Electron-withdrawing substituents can facilitate reduction but may broaden reductase susceptibility or reduce dark stability; steric shielding can suppress adventitious reduction while limiting access to flavin-containing active sites [1,6,8]. Nitrobenzyl units are consequently useful modular switches, whereas nitroimidazoles more often exploit oxygen-sensitive one-electron chemistry and retention of partially reduced products [1,3,4,7,8].

Recognition claims should be matched to the level of evidence. Recombinant-enzyme turnover and computational binding models establish catalytic competence or plausible binding modes, not dominance in cells. Product analysis, defined reductase panels, and mechanism-specific interferents are more informative than broad panels of nonreactive ions or metabolites; kinetically orthogonal H_2_S/NTR measurements illustrate this principle [8,26].

### 3.2. Linker Architecture and Chemical Conversion

Linkers determine whether nitroreduction is the terminal reporting event or the first step in a chemical cascade. A cleavable or self-immolative linker can lower pre-activation signal and release a reporter or payload, but the observed response then combines enzymatic reduction, fragmentation, diffusion, and accumulation. Host–guest shielding and hydrolysis-resistant linkers should therefore be evaluated against unprotected controls under serum and competing-nucleophile conditions [27,28,29].

Architecture should be selected according to the desired temporal and spatial readout. Direct reduction preserves molecular connectivity and can more closely track conversion; fragmentation creates products with new diffusion and clearance behavior; covalent capture or assembly improves retention but makes the signal cumulative and dependent on accessible targets. Reversible systems may better follow dynamics, whereas irreversible systems favor sensitivity and persistence. Detailed elimination, cyclization, and payload-release mechanisms are compared in Section 4.1.4.

### 3.3. Output Modules and Performance Determinants

Output modules are an orthogonal design layer: they determine wavelength, localization, retention, and therapeutic function but do not change the underlying chemical architecture. NIR fluorescence, ratiometric readouts, two-photon excitation, photoacoustic imaging, chemiluminescence, and ^19^F NMR address different measurement problems and require modality-specific calibration [11,30,31,32,33,34,35,36,37]. None alone establishes universal imaging depth or absolute quantification.

Targeting ligands, organelle vectors, photosensitizers, gasotransmitter donors, and drug-release modules can solve defined delivery or functional limitations, but each introduces a new determinant of signal, including receptor expression, probe concentration, release yield, local oxygen, or pharmacokinetics [10,38,39,40,41,42,43,44]. The architecture-specific comparisons in Section 4 therefore evaluate whether the added module improves a prespecified endpoint relative to a simpler control.

The recognition, linker, and output modules define the molecular components of a responsive system but do not by themselves describe the sequence of chemical events after reduction. The same nitroaromatic trigger may produce a direct electronic change, initiate bond cleavage, or promote retention and assembly. Section 4 therefore classifies NTR-responsive systems according to reaction architecture and then considers how each chemical event is translated into a measurable output.

## 4. Comparative Reaction Architectures and Signal-Transduction Mechanisms

For mechanistic comparison, NTR-responsive systems are analyzed along three independent axes. The primary axis is the dominant chemical event following nitroreduction: direct electronic reconfiguration, reduction-triggered fragmentation, or reduction-triggered retention, covalent capture, or assembly. The second axis is signal transduction, including PET, ICT, FRET, AIE, ratiometric fluorescence, chemiluminescence, photoacoustic imaging, and ^19^F NMR. The third axis is the biological application context. Separating these dimensions prevents optical modalities or disease models from being misclassified as distinct reaction mechanisms and allows systems to be compared according to the analytical problem they address (Figure 8).

### 4.1. Reduction-Triggered Fragmentation Systems

Reduction-triggered fragmentation systems connect a nitroaromatic trigger to a reporter or payload through a cleavable linker. Reduction to a hydroxylamine or amine initiates 1,6-elimination, cyclization, decarboxylation, or related fragmentation. The architecture is modular and can suppress pre-activation signal or release therapeutic cargo, but the final output integrates enzymatic turnover, chemical fragmentation, product diffusion, and accumulation.

#### 4.1.1. PET-Controlled Turn-On Systems

In PET-controlled systems, the nitroaromatic trigger acts as an electron acceptor that promotes nonradiative decay. Reduction weakens this acceptor and restores emission. The attainable contrast depends on redox potentials, solvent polarity, protonation, and trigger–reporter geometry; PET is therefore a transduction mechanism rather than a guarantee of low background.

Across the early PET-controlled cleavage systems, dicoumarin and low-background NIR designs (Probes 1 and 2) established cellular, whole-animal, and surgical-guidance feasibility [45,46] (Figure 9). Their reported detection limits were not directly rankable because enzyme concentration, cofactor conditions, solvent composition, optical windows, and biological endpoints differ. More transferable criteria include dark stability, fold change at matched conversion, product identity, and contrast after accounting for delivery and clearance. The resulting design rule was that analytical sensitivity and biological detectability should be reported separately: a favorable solution-phase response may not survive uptake, compartmentalization, or clearance, whereas targeted accumulation could improve lesion contrast without changing intrinsic reduction kinetics.

When PET control was coupled to AIE or phototherapy, as in Probes 3 and 4, nitroreduction, aggregation, intersystem crossing, and ROS generation became interdependent [26,27] (Figure 10). This coupling could preserve emission and photochemical activity in aggregated states, but a stronger cellular signal might reflect greater conversion, greater aggregation, or both. Matched measurements of chemical conversion, aggregate state, ROS yield, and oxygen dependence were therefore required before an apparent therapeutic gain could be assigned to the activation mechanism.

Targeting modules introduced a different trade-off and carbonic-anhydrase- and folate-directed examples (including Probe 5) could increase tumor accumulation, but the observed contrast then reflected both receptor expression and nitroreduction [28]. Non-targeted analogues, receptor blocking or receptor-negative cells, matched tissue concentrations, and ex vivo product analysis were required to separate delivery from chemical activation (Table 3).

#### 4.1.2. ICT-Controlled Spectral Responses

ICT-controlled systems translate nitro-to-amine conversion into a change in donor–acceptor strength and excited-state charge distribution. They can provide wavelength shifts or ratiometric outputs, but the same transition is sensitive to polarity, protonation, protein binding, aggregation, and restricted rotation.

The representative NIR ICT system (Probe 6) demonstrated oxygen-dependent contrast in cells and tumor-bearing mice [29], but longer-wavelength emission alone did not establish general deep-tissue performance (Figure 11). Across ICT designs, spectral stability should be tested against polarity, protein binding, aggregation, probe concentration, and tissue attenuation, and product formation should be confirmed before a specific enzyme is assigned.

Viscosity-amplified and dual-channel mitophagy systems showed that environmental sensitivity can be either an intended second input or an uncontrolled confounder [43,44,47]. Conversion-based calibration and channel-orthogonality controls would therefore be more informative than comparisons of raw intensity among cellular states.

Overall, mechanism-specific ambiguity remains important. Nitroaromatic triggers might undergo convergent reduction pathways, and ring-opening reporters can be shifted by pH, nucleophiles, or protein binding. Kinetically orthogonal H_2_S/NTR measurements, isolated-product spectra, and structure-resolving product analysis were therefore needed to distinguish true conversion from environment-dependent emission [28].

#### 4.1.3. FRET-Based and Ratiometric Systems

FRET-based systems also utilized nitroreduction-triggered changes in donor–acceptor distance, orientation, or spectral coupling to alter an emission ratio. Internal referencing could reduce common-mode variation in probe concentration and illumination, but direct acceptor excitation, donor bleed-through, unequal quantum yields, detector response, and differential photobleaching still required correction.

The spheroid-imaging system (Probe 8) suggested the practical value of a ratiometric readout in heterogeneous three-dimensional samples [11] (Figure 12). Its principal advantage was comparative robustness rather than absolute quantification; the ratio still required calibration against chemical conversion and correction for wavelength-dependent attenuation.

Multimodal and therapy-coupled variants extend the same design logic. Probe 9 combined fluorescence and photoacoustic outputs, whereas Probe 10 links FRET-regulated fluorescence to subcellular localization and phototherapy [38] (Figure 13). These additions solved different problems—depth, localization, or treatment activation—but also introduce modality-specific concentration, aggregation, absorption, and ROS dependencies.

A credible comparison therefore involved a common conversion-dependent calibration, tests in protein-rich media, channel cross-talk correction, and comparison with ex vivo tissue concentration or product measurements (Table 4). Concordant images alone could reflect shared delivery rather than agreement between physical readouts.

#### 4.1.4. Self-Immolative Release Systems

Self-immolative systems translate nitroreduction into spontaneous linker fragmentation and are most useful when the active reporter or therapeutic payload must be physically separated from the trigger. Their modularity can suppress background and accommodate diverse cargo, but response time includes both enzymatic reduction and subsequent chemical release.

Mechanistically, para-aminobenzyl-type spacers commonly undergo 1,6-elimination, whereas ortho-substituted relays can favor intramolecular cyclization; carbonate, carbamate, and ether linkages release different classes of payload with different kinetics. Aniline basicity, spacer substitution, leaving-group ability, pH, and hydrolytic stability control the rate and product distribution [1,27,28,29].

The principal consideration should be interpretability, although enzyme turnover, fragmentation, diffusion, and secondary chemistry are convolved in the final signal, and incomplete release could generate fluorescent intermediates or reactive by-products. Product mapping, mass balance, release yield, and dark or normoxic stability should therefore accompany optical kinetics.

Payload identity also determined the next validation layer: H_2_S donors required direct quantification of carbonyl sulfide/H_2_S formation and carbonic-anhydrase dependence; proteolysis-targeting chimera (PROTAC) systems require confirmation of restored ternary-complex formation and target degradation; and amine-delivery systems require measurements of payload release and localization [31,33,49]. A common fluorescence trace could not substitute for these payload-specific endpoints.

Representative systems span dual-readout mitophagy imaging, mitochondrial amine delivery, covalent labeling, and gasotransmitter release (Probes 12–15) [30,31,32,33] (Figure 14). These examples shared the same fragmentation architecture but answer different biological questions; comparisons should therefore focus on release efficiency, perturbation of the targeted compartment, accessible labeling targets, and nonspecific hydrolytic or thiol activation rather than on a single detection-limit ranking.

At the nanoplatform level, fragmentation has been coupled to metabolic remodeling and positive feedback [47,50]. Amplification could reveal weak initial activity but breaks a simple proportional relationship between baseline nitroreduction and final signal. Dose–response analysis, temporal modeling, product verification, and off-target measurements were required to separate initial activation from feedback-driven accumulation (Table 5).

### 4.2. Direct-Reduction-Type Systems

Direct-reduction systems incorporate the nitro group into the reporter or a conjugated substituent without a cleavable spacer. Reduction directly changes electronic structure, allowing compact designs and potentially rapid switching, but offering less modular control over background, retention, and payload release than fragmentation-based systems. Across mammalian examples, viscosity amplification, targeting ligands, organelle localization, and reversible binding revealed the central trade-off of direct-response designs: the same compact scaffold that coupled reduction efficiently to fluorescence also coupled the output to local polarity, viscosity, binding, and concentration [34,39,40,41] (Figure 15).

The design advantage is therefore greatest when rapid electronic switching is the stated endpoint. When environmental amplification or targeting is added, matched controls must separate conversion from rheology, receptor expression, biodistribution, or organelle state.

Phototherapy-coupled and reversible systems extended the architecture in different directions. C2′-aryl cyanine substitution could tune spectral switching and photosensitizer activation, whereas reversible binding may improve temporal tracking [40,41]. Neither should be ranked against solution-phase reporters by detection limit alone because organelle localization, long incubation, binding equilibrium, and enzyme turnover contribute to the observed response.

Native microbial applications shared the direct-reduction architecture but not the biological meaning of mammalian hypoxia-associated imaging. Rapid bacterial responses and infection–tumor discrimination (Probes 20 and 21) depended on organism burden, growth phase, oxygenation, and the density of reductases distributed across viable cells [24,42] (Figure 16).

Accordingly, microbial studies should normalize for viable count or biomass and include killed-bacteria, sterile inflammatory, and mammalian-cell controls. An apparent kinetic advantage might otherwise reflect high bacterial burden rather than faster turnover by an individual enzyme.

Organelle-targeted and multimodal direct-response systems required the same decomposition. Colocalization established distribution rather than enzyme identity, while fluorescence and ^19^F NMR report different physical quantities [35,36,39,40,41]. Shared conversion standards, organelle-function controls, and ex vivo product analysis was needed before agreement between channels is interpreted as quantitative validation (Table 6).

### 4.3. Reduction-Triggered Retention, Covalent Capture, and Assembly Systems

Reduction-triggered retention systems couple nitroreduction to covalent labeling, aggregation, phase transition, or in situ immobilization. The secondary event can improve signal persistence and reduce washout, but the measured intensity then depends on reduction, capture or assembly kinetics, accessible target abundance, local concentration, and clearance. These systems are therefore well suited to spatial retention but not to instantaneous enzyme-rate measurements.

Probe retention affects the temporal and spatial meaning of the measurement. A freely diffusible product might provide a much closer temporal relationship to conversion but could blur spatial boundaries, whereas covalent capture, phase transition, or self-assembly preserved location at the cost of kinetic interpretability. Covalent assembly and liquid-to-solid transition systems demonstrated how reduction can create persistent two-photon or AIE signals in tumor tissue [37,43]. The retained material should be characterized for target identity, assembly morphology, washout, and reversibility, because each variable contributes to image persistence.

Persistent local retention may provide therapeutic advantages. The self-immobilizing photosensitizer remained in the activated region and extended the irradiation window [44], while the metabolic reporter covalently labeled pathological hypoxic cells [32]. These strategies solve different problems—drug residence versus lineage or spatial mapping—and should not be merged into a single performance category. Long-term histology and clearance are particularly important when the retained species is chemically reactive.

Representative retention systems use covalent assembly, two-photon reporters, or protein labeling (Probes 24–26) to preserve the location of activation [37,44,51] (Figure 17 and Figure 18). These strategies improved tissue persistence and boundary definition, but the final intensity integrated reduction, capture or assembly kinetics, accessible target abundance, washout, and clearance. Target identity, assembly morphology, reversibility, biodistribution, and long-term histology are therefore more informative than instantaneous enzyme-rate measurements (Table 7).

### 4.4. Cross-Architecture Design Rules

Architecture selection should follow the intended measurement rather than a general preference for molecular complexity. Direct electronic reconfiguration is most suitable for compact and potentially dynamic readouts; fragmentation is preferred when low background or cargo release is required; and retention, capture, or assembly is preferred when spatial persistence is more important than instantaneous kinetics (Table 8).

Across all three architectures, the optical modality and biological model should be treated as modifiers of interpretation rather than as evidence of a superior mechanism. Added modules are justified only when they improve a prespecified endpoint and when the new dependency they introduce is tested with matched simpler controls.

Reaction architecture determines activation kinetics, background stability, product identity, and signal persistence. These properties become especially important in biological applications, where transport, enzyme abundance, oxygenation, clearance, and treatment timing can influence the observed response. Section 5 consequently evaluates how these chemical designs are used in multi-input sensing and activatable therapy and what evidence is required to support biological or translational conclusions.

## 5. From Single-Input Sensing to Logic-Gated Imaging and Activatable Therapy

The application-level progression of NTR-responsive systems is not a simple increase in module number. Single-input probes remain appropriate for mechanistic studies when reduction specificity is controlled. A second input is justified only when it addresses a defined background pathway, and a therapeutic module is justified only when signal activation is related quantitatively to payload release or photochemical output. The sections below therefore compare the problem solved by each added module with the new variables it introduces (Figure 19).

### 5.1. Multi-Input Recognition for Higher-Fidelity Imaging

A logic gate improved specificity only with respect to a defined background mechanism. Acidic pH might distinguish extracellular or endosomal compartments, GSH might report a reductive intracellular environment, and a second enzyme could restrict activation to a particular cell state. The gain in specificity depended on the distributions and thresholds of both inputs, not on the formal AND symbol. In a heterogeneous lesion, a stringent gate would suppress clinically relevant regions that express one input weakly or at a different time.

Sequential gating can impose an ordered activation sequence that reduces specific sources of background during disease-associated imaging. If pH-dependent disassembly controlled delivery before nitroreduction, the measured lag would include transport and nanostructure remodeling; if two enzymes unmasked a reporter in either order, intermediate states might accumulate. Complete truth-table experiments should therefore be complemented by kinetic traces, matched tissue concentrations, and single-lock analogues. These controls revealed whether the second input reduced chemical background or simply reduced the amount of probe reaching the active state.

#### 5.1.1. Sequential pH/NTR Gating

Probe 11 employed acid-sensitive nanomicelle disassembly before nitroreduction-dependent fluorescence activation (Figure 20) [48]. The design addressed premature activation by requiring two conditions, but the reported output integrates micelle disassembly, probe release, nitroreduction, and tissue delivery. Its logical selectivity should therefore be supported by pH-only, NTR-only, and fully activated controls across the pH range observed in heterogeneous tumors.

#### 5.1.2. GSH/NTR Redox Gating and Scope Boundaries

Where GSH was used as a second input, the NTR-dependent chemical step must remain explicit. Co-localization of high GSH and nitro-reducing activity might improve conditional activation, but GSH concentration varied within and among tumors and could create false negatives when the threshold is too stringent [53]. GSH-only, pH/GSH, or general redox-responsive nanomedicines without an NTR-dependent step were mechanistically distinct and excluded from the principal comparison.

GSH gating was mechanistically useful only when its role in the activation sequence was explicit. High intracellular GSH could unmask a reporter, alter nanoparticle structure, or change redox balance, but these processes were not equivalent. The current review retains only platforms in which nitroreduction remained a necessary input and excludes GSH-only or pH/GSH systems from the main comparison. This boundary prevented the broad tumor-microenvironment literature from being presented as evidence for NTR-responsive design.

#### 5.1.3. Enzyme/NTR and Gates for Image-Guided Intervention

The DHP platform employed GGT and nitroreduction as sequential activation inputs before near-infrared fluorescence and combined photodynamic–photothermal output [54] (Figure 21). Its central contribution was conditional activation rather than the mere addition of two therapeutic modes. A convincing analysis therefore requires every single-input state, enzyme-negative or inhibitor-treated controls, time-resolved activation, and comparison with a single-lock analogue. In heterogeneous or necrotic tissue, the gate may fail because one input falls below threshold even when the lesion is clinically relevant; margin contrast and false-negative performance should consequently be reported together.

#### 5.1.4. Feedback Amplification: Sensitivity Gain Versus Interpretability Loss

Positive-feedback platforms coupled nitroreduction to metabolic remodeling or catalytic amplification [47,50]. They can reveal regions with weak initial activity, but the final signal reports the complete feedback circuit rather than baseline nitro-reducing activity. Dose–response and time-resolved experiments are required to distinguish primary chemical activation from feedback-driven accumulation and to determine whether amplification improves lesion detection without increasing off-target activation.

The glucose-oxidase-inspired amplification system and the hypoxia nano-amplifier illustrated two routes to sensitivity enhancement [47,50]. In both cases, initial activation changed the microenvironment or catalytic capacity in a way that promoted further response. This was useful for weakly hypoxic lesions, but it broke a simple proportional relationship between starting reductase activity and final signal. A mechanistically informative study should quantify the unamplified baseline, the amplification gain, the time at which feedback becomes dominant, and the effect on off-target tissues.

### 5.2. NTR-Responsive Theranostic Platforms

Theranostic systems combine target-dependent signal generation with drug release or phototherapy. Their value lies in reporting where and when treatment is activated. Demonstrating fluorescence alone is insufficient: the release fraction, photophysical state, therapeutic dose, off-target activation, and relationship between signal and treatment effect must also be quantified.

For therapy-coupled designs, the phrase ‘self-reporting’ should be reserved for systems in which the reporter has been calibrated against the therapeutic event. Fluorescence can precede complete drug release, persist after a diffusible payload has left the site, or respond differently from a photochemical pathway. The necessary validation depends on the treatment: release fraction and active-drug concentration for prodrugs, singlet-oxygen or radical yield for PDT, photothermal conversion for PTT, and target degradation for PROTACs. Tumor growth inhibition alone cannot resolve these intermediate steps.

Dose–response relationships should be established at more than one level. Administered dose determines systemic exposure; intratumoral concentration determines the amount available for activation; chemical conversion determines active payload; and pharmacodynamic response depends on target susceptibility. A fluorescence or chemiluminescence signal may correlate with any of these variables without uniquely reporting the others. Serial imaging, direct payload analysis, and exposure-matched inactive controls are needed to identify which link in the chain is being measured.

#### 5.2.1. NTR-Triggered Drug Release and Chemiluminescence-Guided Activation

NTR-triggered cleavage has been used to release cytotoxic or bioactive payloads from self-immolative constructs [52,55,56]. Probe 27 coupled camptothecin release to chemiluminescence, allowing the activation process to be followed without external excitation (Figure 22) [52]. The relevant endpoint was not fluorescence or chemiluminescence fold-change alone, but the relationship among chemical conversion, released dose, tumor exposure, therapeutic effect, and systemic toxicity.

The chemiluminescent camptothecin prodrug provided a particularly clear test of signal–dose coupling because the same activation cascade generates light and releases the drug [52]. Its value depended on demonstrating that chemiluminescence tracked conversion across concentrations, biological matrices, and time, rather than merely co-occurring with therapeutic response. Evodiamine-based agents and NTR-gated mesoporous carriers extended the release concept to different payload and formulation architectures [55,56]. Across these systems, release yield, active-drug exposure, leakage under normoxia, and systemic toxicity should be compared at matched administered dose.

NTR-responsive payloads also extended beyond conventional cytotoxics. Gasotransmitter donors and caged protein degraders illustrated how the same chemical trigger could regulate short-lived signaling molecules or catalytic pharmacology [33,49]. Their evaluation could not be reduced to fluorescence fold change: H_2_S production, target-protein loss, downstream pathway modulation, and rescue controls were required to establish that the intended therapeutic mechanism was engaged.

For the chemiluminescent prodrug, photon output relied on substrate availability, reaction efficiency, and biodistribution in addition to nitroreduction-dependent conversion. A calibration linking light output to camptothecin release was therefore necessary before the signal could be interpreted as a dose surrogate. Feedback-amplification platforms are considered separately in Section 5.1.4.

#### 5.2.2. Activatable Photodynamic Therapy

NTR-responsive photosensitizers were designed to suppress fluorescence and photosensitization before reduction and to restore both functions after activation [26,57]. This coupling was attractive because the optical signal could indicate where photochemistry was permitted, yet fluorescence recovery was not a surrogate for singlet-oxygen dose. The studies represented by the mitochondrial, lysosomal, and AIE photosensitizers differ in localization, aggregation state, and oxygen dependence; they should therefore be compared through dark toxicity, phototoxicity, ROS or singlet-oxygen yield, irradiation conditions, and the correspondence between chemical conversion and therapeutic output.

Photodynamic performance was constrained by the same hypoxia that motivated NTR activation in activatable photodynamic therapy. Type I photosensitizers and aggregation-engineered systems might retain activity at lower oxygen than conventional Type II sensitizers, but they still required explicit oxygen-dependent ROS measurements [26,57]. Organelle targeting could increase local damage yet also perturbed the compartment used as a biological readout [57]. The most persuasive studies therefore separated localization, reduction, fluorescence recovery, ROS production, cell-death pathway, and in vivo clearance rather than presenting them as one activation endpoint. NFh-NTR provides a representative example: NTR activation releases the photosensitizer NFh-NMe-2, restores fluorescence, and enables tumor-cell killing after 808 nm irradiation (Figure 23) [58].

The distinction between Type I and Type II photochemistry was relevant but should not be used as a promotional label. Radical-generating pathways might be less oxygen-demanding than singlet-oxygen production, yet their efficiency still depended on electron donors, local oxygen, aggregation, and competing quenchers. Comparative experiments under controlled oxygen concentrations were required to show that an NTR-activated photosensitizer retains therapeutic output in the hypoxic range where activation occurs [26,57].

#### 5.2.3. Photothermal and Multimodal Therapy

Direct nitroreduction-gated photothermal systems remained less common than fluorescence or photodynamic platforms. The GGT/nitroreduction dual-lock provided one example in which sequential activation changed both the imaging state and the balance between photodynamic and photothermal output [54], whereas self-immobilizing systems prolonged local exposure after activation [44]. Photothermal conversion efficiency should be measured before and after activation under matched concentration, formulation, irradiation power density, and exposure time. A temperature rise by itself did not establish nitroreduction specificity, because nanoparticle accumulation, aggregation, and tissue perfusion could produce the same endpoint.

Across chemotherapy, PDT, and PTT platforms, the optical signal, chemical conversion, and delivered therapeutic dose were related but not interchangeable. Linker stability, incomplete conversion, diffusion, aggregation, and oxygen consumption could uncouple imaging from treatment. Theranostic claims are strongest when fluorescence, photoacoustic, chemiluminescent, or thermal output is calibrated against payload release or ROS generation and when simpler single-function controls are included.

### 5.3. Biological Validation and Translational Evaluation in Complex Models

Validation should proceed at three linked levels. At the chemical level, spectral change should be connected to identified reduction products, cleavage yield, and dark or normoxic stability. At the enzymatic level, the source and concentration of the reductase, NADH or NADPH conditions, oxygen tension, competing enzymes, and inhibitor or genetic controls should be stated. At the imaging level, calibration, dose, timing, tissue attenuation, and analysis procedures determine whether a signal can be compared across samples. A cuvette response therefore cannot be assumed to represent the same biochemical quantity in vivo.

A practical reporting sequence began with chemistry and ended with biological inference: First, identify the reduced or released product by high-performance liquid chromatography (HPLC), liquid chromatography–mass spectrometry (LC–MS), NMR, or another structure-resolving method. Second, establish turnover with a named enzyme preparation and report cofactor and oxygen conditions. Third, test plausible competing reductases or use perturbations when a specific endogenous enzyme is claimed. Only then should cellular localization, lesion contrast, or therapeutic activity be interpreted. This sequence prevented an attractive image from becoming the primary evidence for an unverified reaction mechanism.

Quantitative claims require an explicit calibration model. A ratio could reduce common-mode intensity artifacts but did not remove wavelength-dependent attenuation; photoacoustic amplitude depended on absorption and heat conversion; chemiluminescence relied on substrate supply and reaction quantum yield; and ^19^F NMR depended on chemical shift, relaxation, and local concentration [11,30,35,37]. Studies should report the calibration range, residual error, biological replicates, and uncertainty associated with translating the analytical signal into conversion or activity.

Statistical reporting is an essential component of quantitative validation. Biological images should be supported by predefined regions of interest, blinded or automated segmentation where feasible, biological rather than only technical replicates, and effect estimates with uncertainty. For image-guided surgery, sensitivity, specificity, and margin assessment are more informative than representative fluorescence photographs; for treatment studies, longitudinal tumor response and toxicity should be analyzed alongside endpoint volume. These practices make the evidence more transferable without imposing a formal evidence-rating scheme.

Targeted probes evaluated in orthotopic or otherwise difficult-to-image tumors can improve anatomical relevance [28,35,46], but apparent tumor selectivity should be decomposed into delivery, ligand-dependent accumulation, and nitroreduction-dependent activation. Receptor-negative tumors, nonmalignant inflammatory lesions, blocking experiments, matched non-targeted analogues, and quantitative histology are required to determine which component produces the observed contrast. Without these controls, an increase in lesion-to-background ratio might reflect pharmacokinetics rather than a more selective chemical reaction.

Non-oncological models provided useful stress tests of biological interpretation. Ulcerative colitis and sepsis-associated lung injury involved hypoxia together with inflammation, microbial metabolism, altered perfusion, and changing immune-cell composition [59,60,61,62]. Infection studies might increase organism burden and disturb growth state, whereas engineered zebrafish systems introduced a defined bacterial NTR transgene [20,21,22,23,24]. These models should be discussed separately because the same optical chemistry answered different biological questions in each setting.

Zebrafish provided optical access for assessing uptake, developmental effects, and spatial activation [22,37,51]. These models were informative for distribution and tissue-level response, but they should not be extrapolated directly to mammalian pharmacokinetics because absorption, metabolism, immune clearance, and optical path length differ.

Host–guest encapsulation and hydrolysis-resistant shielding illustrated how formulation can modify substrate access and chemical stability [63,64]. Their benefit should be demonstrated under serum, competing nucleophile, and prolonged-incubation conditions with an explicit unprotected-probe control; otherwise, improved in vivo contrast cannot be assigned specifically to the protective strategy.

Across complex models, the most informative studies combined product confirmation, model-specific enzyme attribution, independent hypoxia or reductase measurements, calibrated imaging, biodistribution, histology, and safety. Pharmacokinetics, repeated-dose toxicity, formulation stability, and synthesis reproducibility were prerequisites for translational evaluation, but the current literature predominantly supported preclinical proof of concept rather than readiness for human use.

Human tissue imaging occupied an intermediate evidentiary position. Fresh or archived specimens could demonstrate that a probe responds in clinically derived material, as shown by two-photon and covalent-assembly studies [37,51], but ex vivo activation did not establish in vivo delivery, safety, or diagnostic performance. Claims should specify whether the tissue study evaluated endogenous activity, externally added enzyme, histological correlation, or disease classification, and should avoid treating the presence of human tissue as evidence of clinical translation.

Manufacturability was rarely discussed at the probe-discovery stage, yet it determined whether a promising scaffold could advance. Relevant considerations included synthetic step count, stereochemical or batch complexity, purification, formulation, serum stability, storage, scale-up, and reproducibility of nanoparticle or aggregate state. These parameters did not need to dominate a mechanistic review, but acknowledging them prevents ‘translational potential’ from being inferred solely from a mouse image.

## 6. Conclusions and Future Perspectives

NTR-responsive systems now include direct-response, cleavage-based, retention-enhanced, logic-gated, and therapy-coupled designs. Their common chemical input is nitroaromatic reduction, but the biological meaning of the output depends on the activating reductases, oxygen and cofactor conditions, probe delivery, linker or assembly kinetics, and clearance. The term “NTR-responsive” should therefore describe an experimentally defined activation context rather than imply a universal mammalian enzyme biomarker. The resulting relationships among the current mechanistic framework, critical limitations, and prospective development directions are summarized in Figure 24.

The relative performance of each reaction architecture depends on the intended analytical or therapeutic application. Direct-response systems may provide rapid optical changes and compact molecular structures, although their signals can be influenced by local polarity, viscosity, or nonspecific interactions. Reduction-triggered cleavage can decrease background signal or release an active payload, but the overall response reflects both enzymatic reduction and subsequent chemical fragmentation. Covalent-capture and assembly-based systems can improve local retention and signal persistence, whereas the measured intensity may additionally depend on target abundance, reaction time, accumulation, and clearance. Similarly, NIR fluorescence, ratiometric fluorescence, two-photon excitation, photoacoustic imaging, chemiluminescence, and ^19^F NMR should be selected according to the specific measurement objective and validated performance. The use of these modalities alone does not establish greater imaging depth, molecular specificity, or quantitative accuracy [11,30,31,32,33,34,35,37].

At the application level, current evidence supports hypoxia-associated preclinical imaging, bacterial discrimination, conditional payload release, photodynamic and photothermal treatment, and multimodal guidance [20,21,22,23,24,26,28,33,35,36,37,38,44,46,49,52,54,55,56,57,59,60,61,62]. These achievements do not yet justify a general claim of precision theranostics or clinical readiness. Each added module should improve a prespecified endpoint relative to a simpler control, and the imaging output should be linked experimentally to chemical conversion, released dose, ROS generation, or thermal deposition rather than interpreted through outcome correlation alone.

The available evidence identifies five methodological priorities: First, enzyme attribution should use defined enzyme panels, product-level confirmation, and inhibitor or genetic perturbation when a specific reductase is claimed. Second, quantitative interpretation should rely on calibrated ratios, validated multimodal relationships, or external standards and should report uncertainty; fluorescence lifetime should be discussed only when an actual lifetime-based platform is included. Third, in vivo studies require pharmacokinetics, biodistribution, repeated-dose safety, and comparison with independent measures of hypoxia or reductase activity. Fourth, synthesis, formulation, stability, and scale-up should be considered early enough to support reproducibility. Fifth, cross-study comparability requires a compact minimum-information set covering probe concentration, enzyme identity and concentration, cofactor, oxygen condition, medium composition, temperature, product verification, response definition, calibration method, imaging dose, analysis pipeline, and biological replicates. Standardized reporting will not make chemically different systems equivalent, but it will distinguish a genuine design advance from a favorable assay condition.

Future progress should be judged by whether a design resolves a defined analytical or therapeutic limitation. Systems that combine chemically verified activation, study-specific biological controls, interpretable outputs, disease-relevant models, and manufacturable structures are the most credible candidates for further preclinical development and, eventually, carefully staged translational assessment.

## Figures and Tables

**Figure 1:**
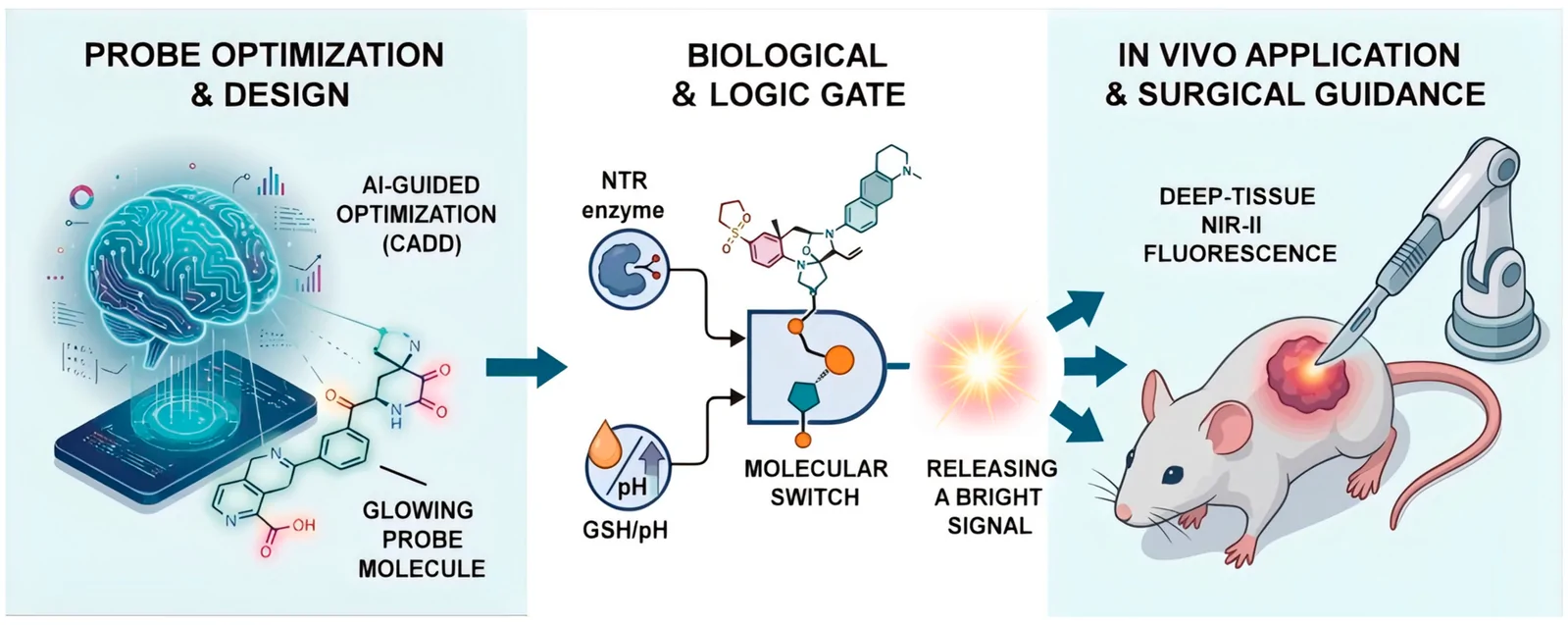
Conceptual roadmap from molecular design and conditional activation to preclinical imaging and therapeutic guidance. AI/CADD and NIR-II integration are presented as prospective development directions rather than established clinical workflows. Author-created schematic.

**Figure 2:**
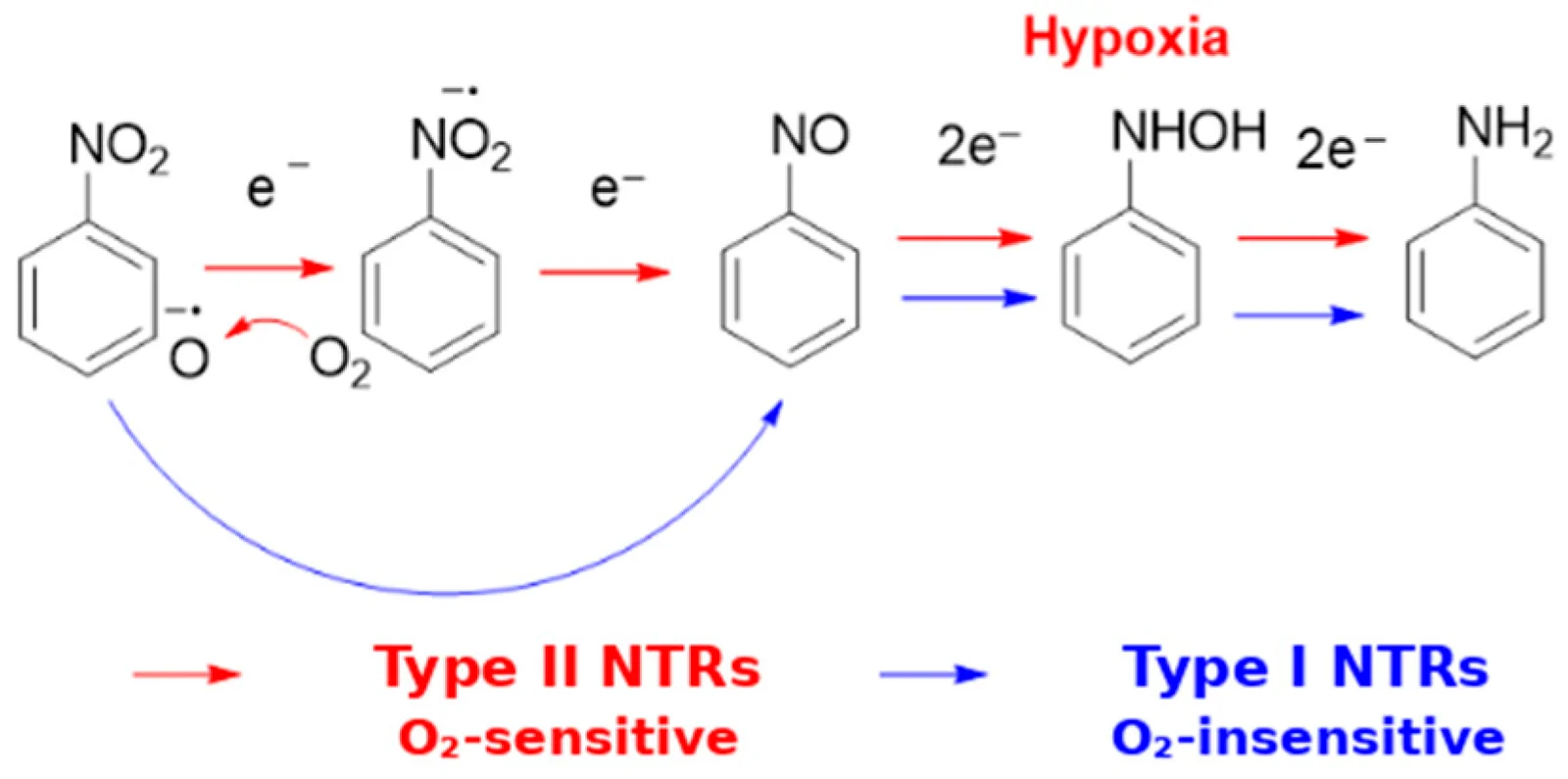
Enzymatic reduction pathways of nitroaromatic compounds. Type I NTRs are oxygen-insensitive two-electron reductases, whereas Type II activity proceeds through oxygen-sensitive one-electron intermediates. Under low oxygen, successive reductions yield nitroso, hydroxylamine, and amine products. Author-created schematic.

**Figure 3:**
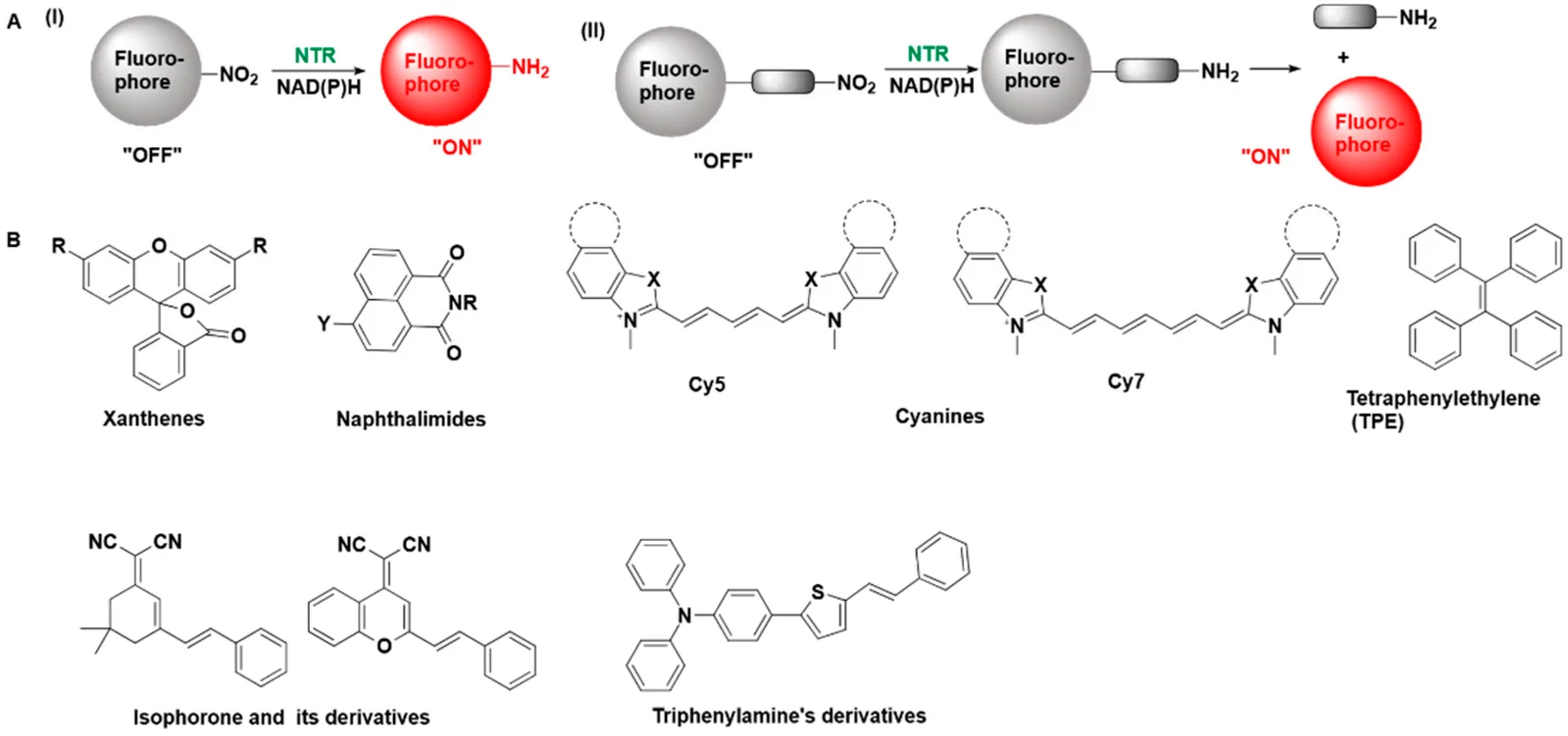
General architecture of NTR-activated turn-on probes (**A**) and representative fluorophore scaffolds used in NTR-responsive systems (**B**). (**A**) (**I**) Direct reduction strategy, in which the nitro group is incorporated into the fluorophore and reduced by NTR in the presence of NADPH to form an amino group, thereby restoring fluorescence; (**II**) Triggered release strategy, in which the nitro-containing moiety acts as a recognition/cleavable unit and, after NTR-mediated reduction, releases the fluorophore to generate a turn-on signal. (**B**) Representative fluorophore scaffolds, including xanthenes, naphthalimides, cyanines (Cy5 and Cy7), tetraphenylethylene (TPE), isophorone derivatives, and triphenylamine derivatives. Author-created schematic.

**Figure 4:**
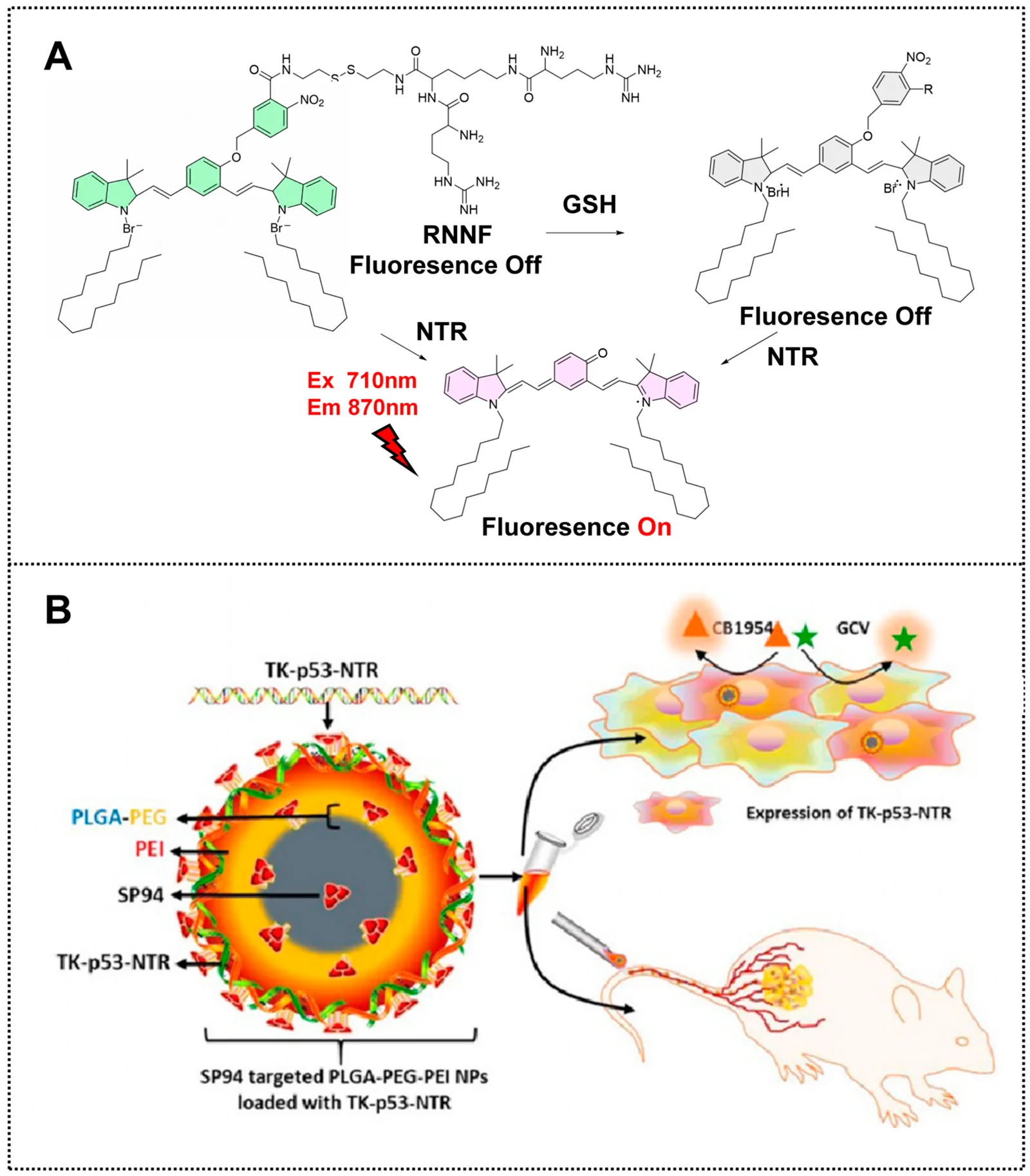
Representative NTR-responsive theranostic systems for hypoxia imaging and cancer therapy. (**A**) Schematic illustration of an NTR-activatable near-infrared fluorescent probe. The probe remains in a fluorescence-off state and is converted into a fluorescence-on form after NTR-mediated bioreduction, producing a signal at Ex 710 nm/Em 870 nm. Glutathione (GSH) is also involved in the activation process. (**B**) Schematic illustration of an SP94-targeted PLGA-PEG-PEI nanoparticle loaded with the TK-p53-NTR gene for tumor-targeted delivery. After transfection, tumor cells express TK-p53-NTR, enabling subsequent activation of the prodrug therapy system and therapeutic response. Copyright 2024, Frontiers in Pharmacology.

**Figure 5:**
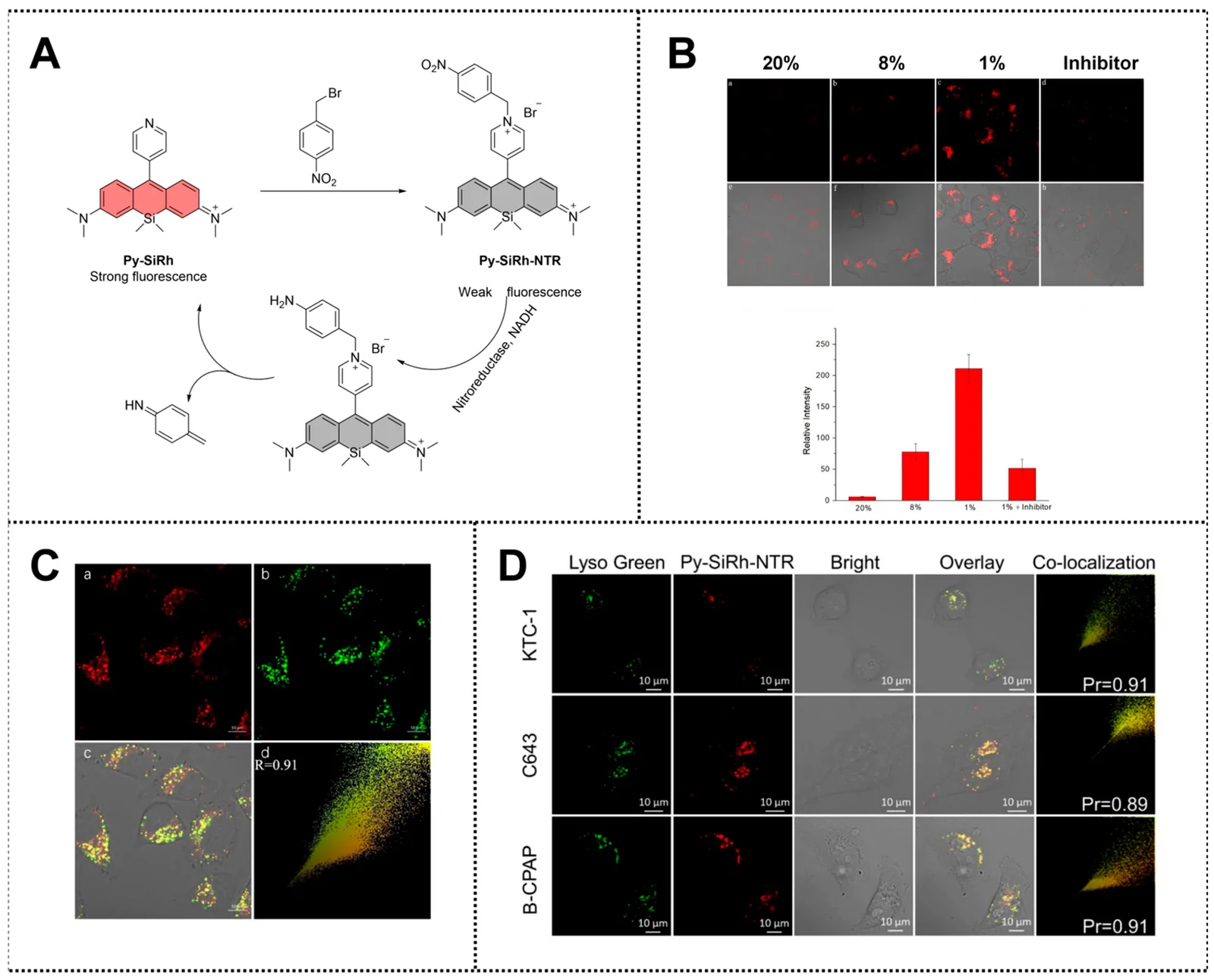
Hypoxia-responsive lysosomal imaging of Py-SiRh-NTR. (**A**) Probe structure and activation mechanism. (**B**) Confocal imaging of HepG2 cells under different oxygen concentrations. (**a**–**d**) The near-IR channel fluorescence image (λex = 633 nm). (**e**–**h**) The merged image of near-IR channel fluorescence image and bright filed imaging. (**C**) Colocalization with LysoTracker Green in HepG2 cells. (**a**) The fluorescence from the **Py-SiRh-NTR**. (**b**) The fluorescence from the LysoTracker green. (**c**) Merged image of images (**a**,**b**). (**d**) Intensity correlation plot between **Py-SiRh-NTR** and LysoTracker green. (**D**) Colocalization in KTC-1, C643, and B-CPAP cells. Copyright 2021, Molecules (MDPI).

**Figure 6:**
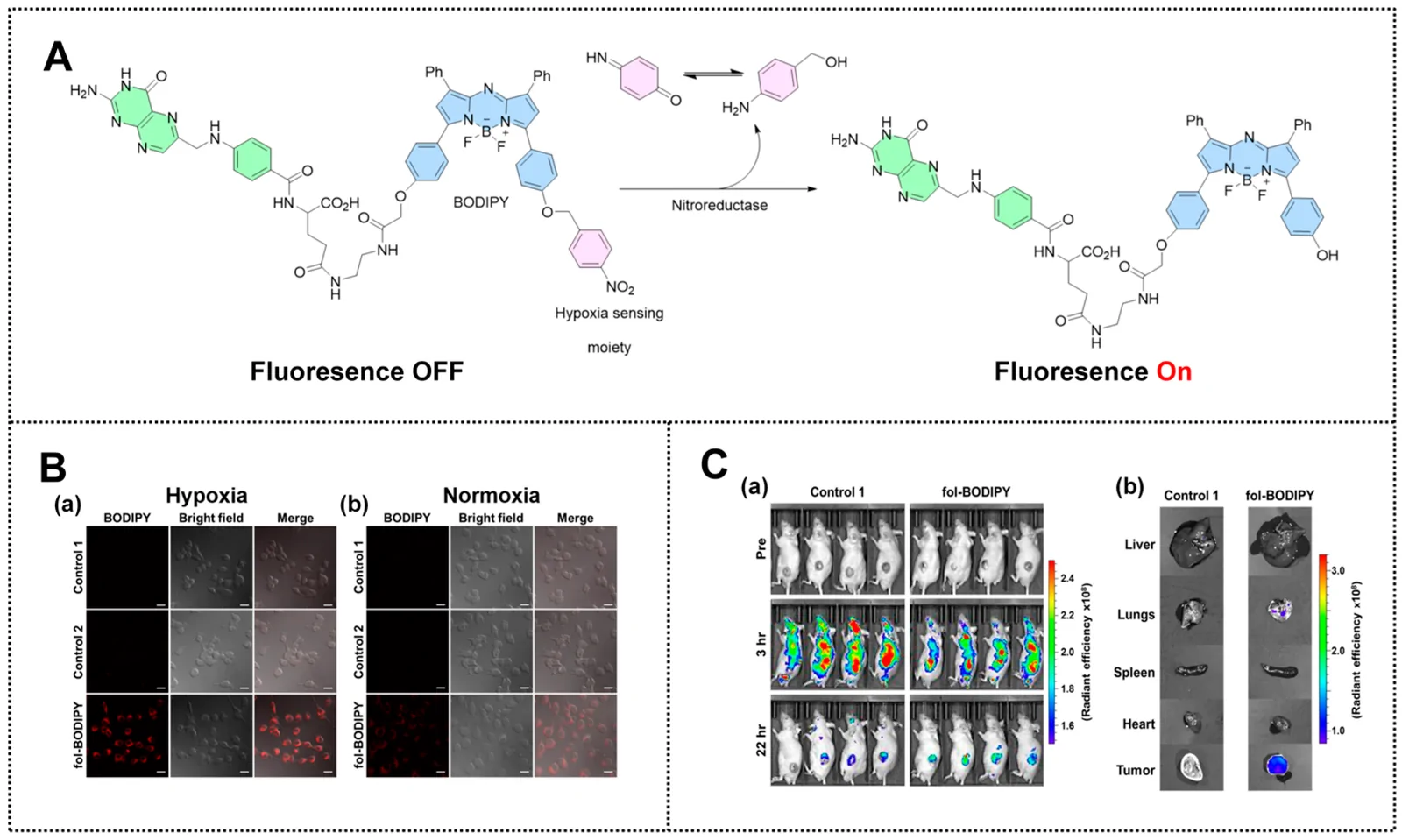
Hypoxia-responsive imaging of fol-BODIPY. (**A**) Schematic illustration of the nitroreductase (NTR)-triggered fluorescence turn-on mechanism of fol-BODIPY under hypoxic conditions. Reduction of the nitro-based hypoxia-sensing moiety by NTR restores the fluorescence of the BODIPY fluorophore. (**B**) Confocal fluorescence imaging of CT26 cells incubated with fol-BODIPY and control probes under different oxygen conditions: (**a**) hypoxia and (**b**) normoxia. Strong fluorescence was observed for fol-BODIPY under hypoxic conditions, whereas weak fluorescence was detected under normoxic conditions and for the control probes. Scale bars: 20 μm. (**C**) In vivo and ex vivo fluorescence imaging of tumor-bearing mice after administration of the probes: (**a**) whole-body fluorescence imaging before injection, and at 3 h and 22 h post-injection; (**b**) ex vivo fluorescence imaging of major organs and tumor tissues, showing preferential accumulation and activation of fol-BODIPY in tumors. Copyright 2021, American Chemical Society.

**Figure 7:**
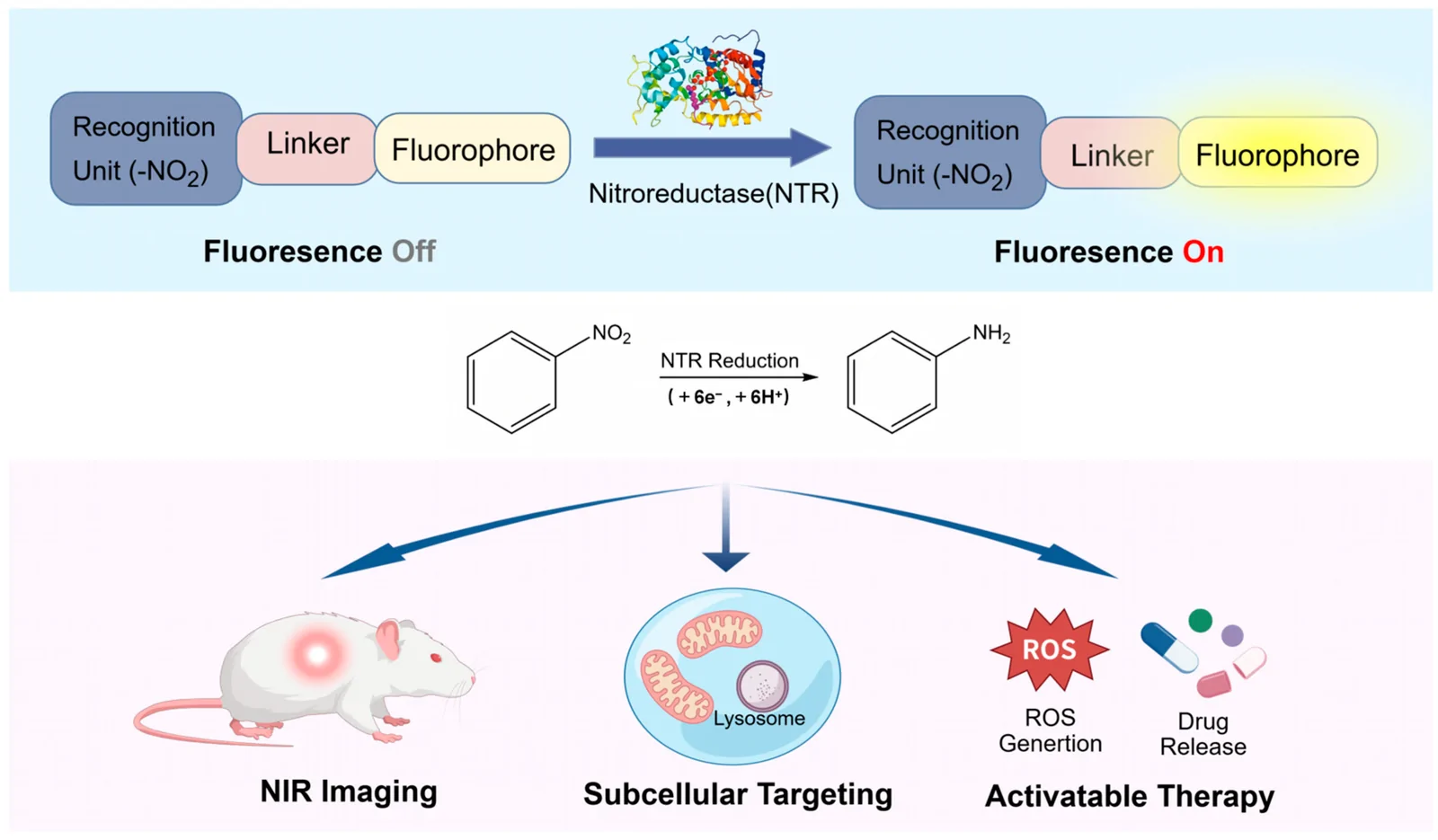
Recognition–linker–output framework for NTR-responsive systems. Nitroreduction may directly regulate fluorophore electronics or initiate cleavage, release, covalent capture, or assembly; output modules then determine spectral mode, localization, retention, and therapeutic function. Created with BioRender.com.

**Figure 8:**
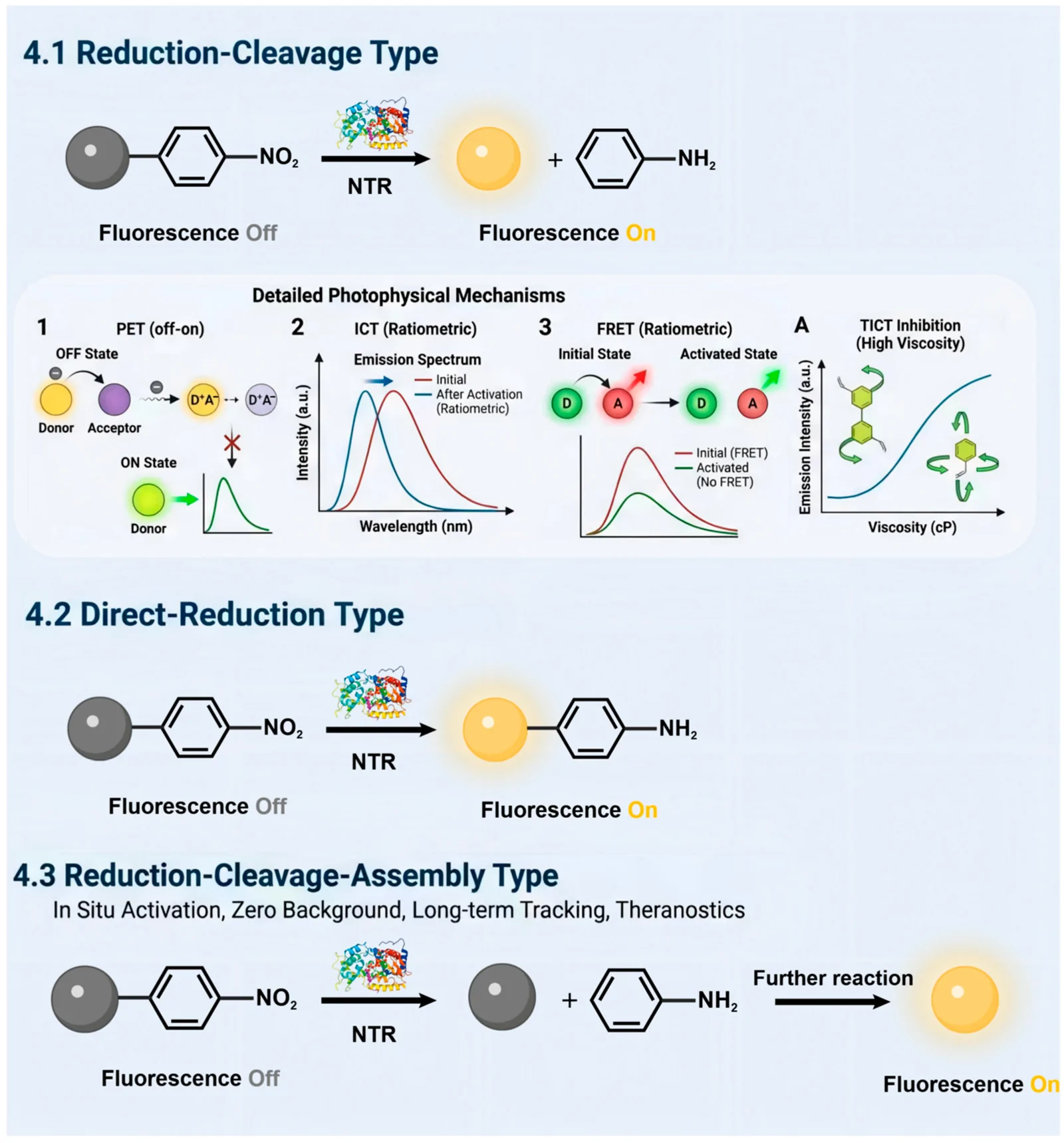
Three dominant chemical architectures of NTR-responsive systems: direct electronic reconfiguration, reduction-triggered fragmentation, and reduction-triggered retention or assembly. PET, ICT, FRET, and related outputs are transduction modes that can operate within these architectures. Author-created schematic.

**Figure 9:**
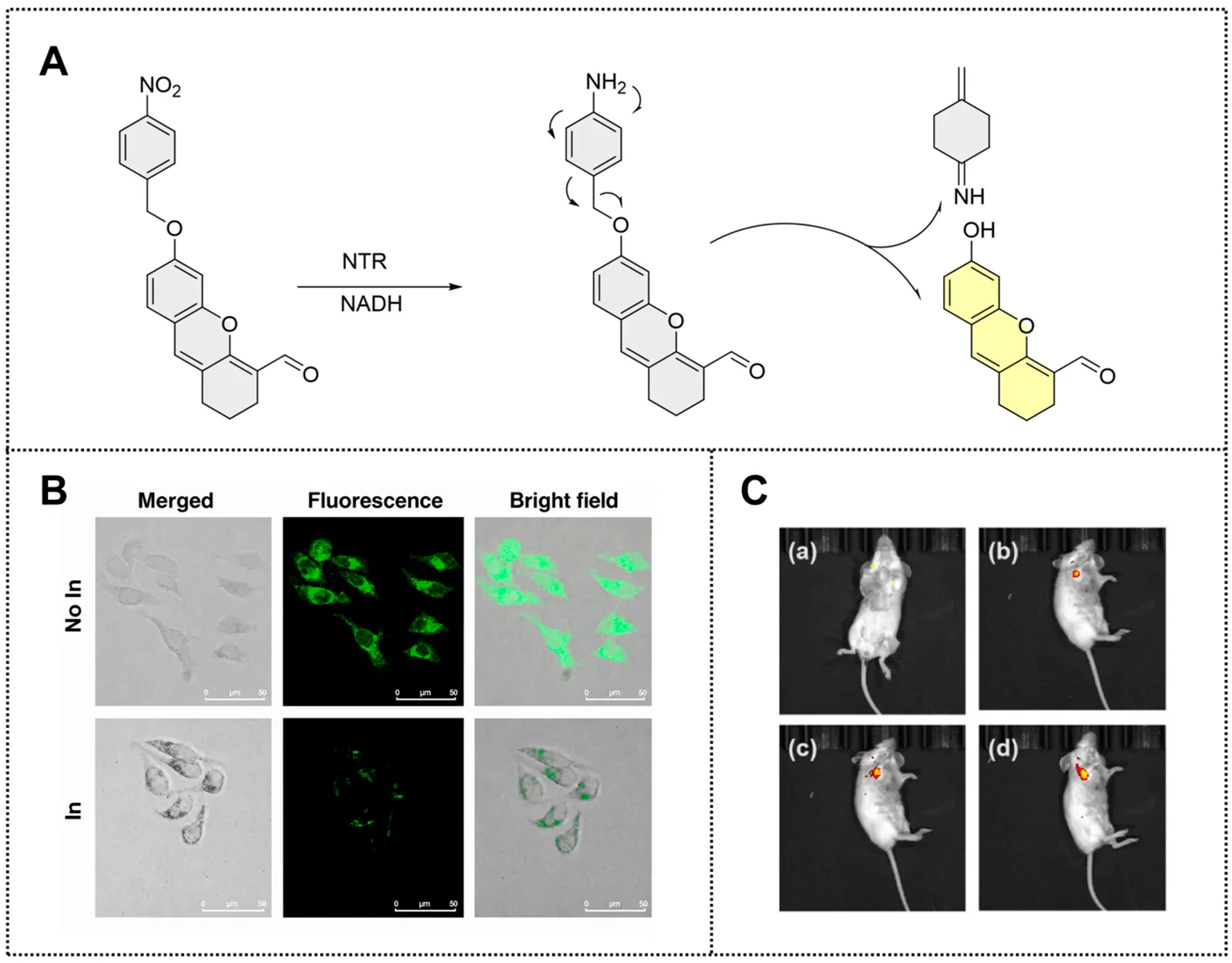
Fluorescence imaging of Probe 1. (**A**) Proposed NTR-triggered activation mechanism of Probe 1 in the presence of NADH, in which reduction of the p-nitrobenzyl group initiates cleavage and releases the fluorescent product. (**B**) Confocal fluorescence imaging of HepG2 cells treated with dicoumarin and Probe 1. (**C**) Whole-body fluorescence imaging of 4T1 tumor-bearing mice: (**a**) saline-treated control; (**b**–**d**) fluorescence images acquired at different time points after injection of F-NTR, showing progressive fluorescence activation in the tumor region. Copyright 2025 Elsevier B.V.

**Figure 10:**
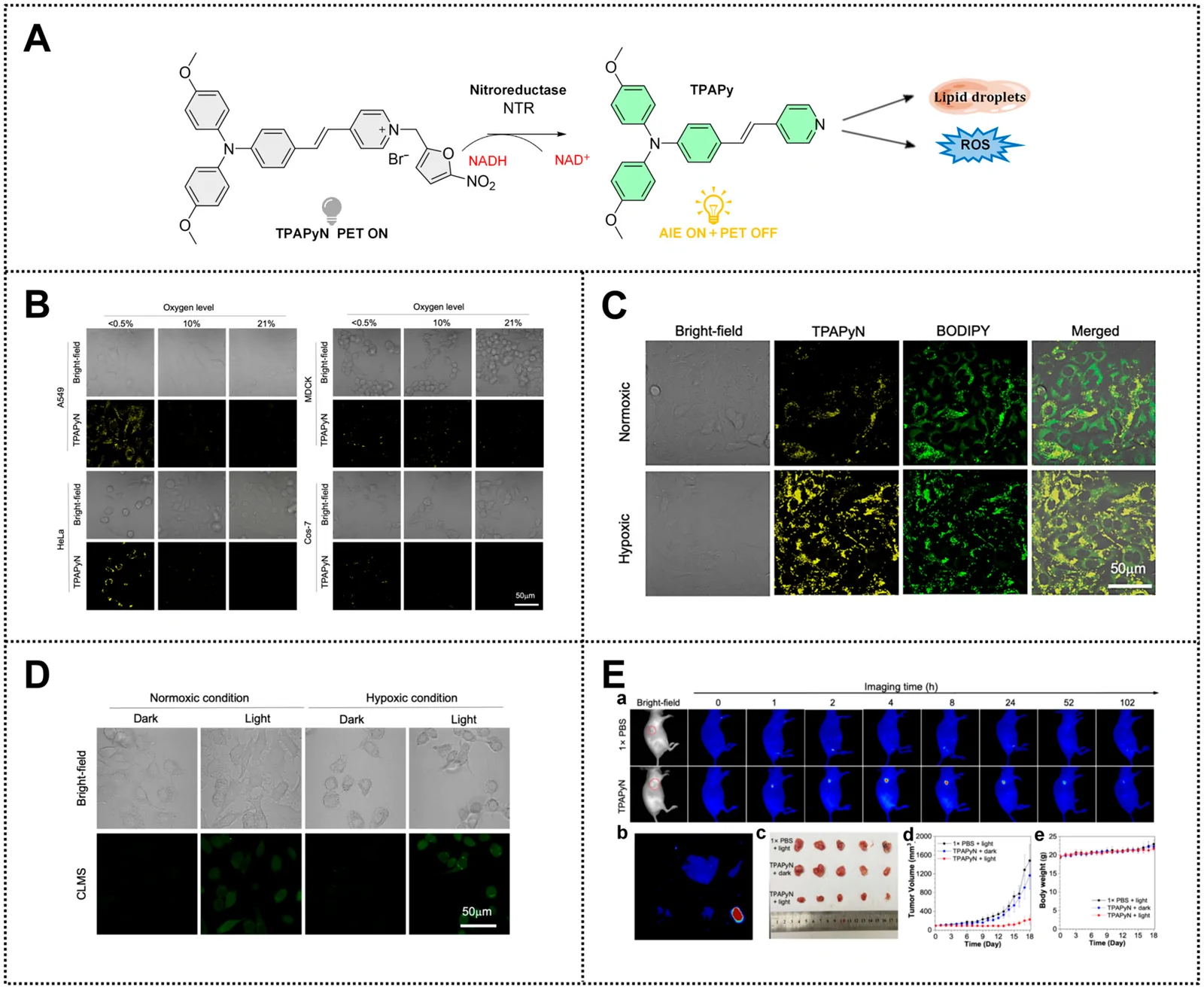
Fluorescence imaging of Probe 3. (**A**) Intracellular NTR-triggered reduction mechanism. (**B**) Cellular imaging across cell lines and oxygen concentrations. (**C**) Photodynamic evaluation in A549 cells and tumor-bearing mice. (**D**) Photodynamic evaluation in CLMS cells. (**E**) In vivo fluorescence imaging and antitumor evaluation in tumor-bearing mice: (**a**) whole-body fluorescence imaging of mice after treatment with PBS or Probe 3 at different post-injection time points; (**b**) ex vivo fluorescence imaging of dissected tumors; (**c**) photographs of excised tumors from different treatment groups; (**d**) tumor growth curves during treatment; and (**e**) body-weight changes of mice, indicating the imaging capability and therapeutic performance of Probe 3. Copyright 2025, American Chemical Society.

**Figure 11:**
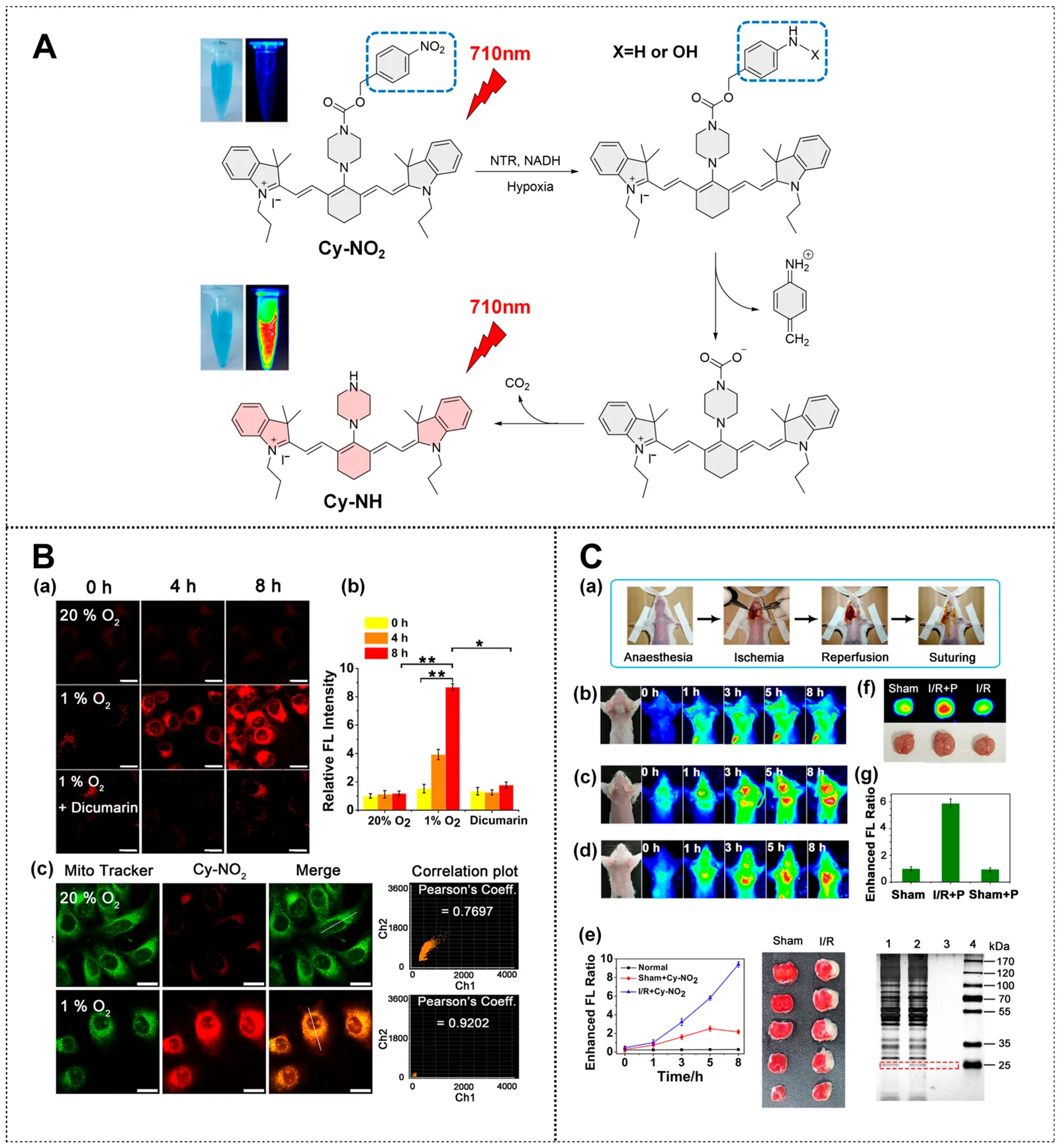
Hypoxia-associated imaging with Probe 6. (**A**) Design and activation mechanism. (**B**) In vitro characterization in A549 cells: (**a**) Confocal fluorescence images of A549 cells incubated with Probe 6; (**b**) The semi-quantitative fluorescent intensity analysis (* *p* < 0.05, ** *p* < 0.01); (**c**) Colocalization imaging of Probe 6 with MitoTracker in A549 cells. Scale bars: 40 μm. (**C**) In vivo imaging in tumor-bearing mice: (**a**) Schematic illustration of the surgical procedure; (**b**) Time-dependent in vivo fluorescence imaging of mice in the Sham + Probe 6 group; (**c**) Time-dependent in vivo fluorescence imaging of mice in the I/R + Probe 6 group; (**d**) Time-dependent in vivo fluorescence imaging of mice in the Sham + P group; (**e**) Enhanced fluorescence ratio as a function of time for different groups, with representative photographs of excised kidneys from the Sham and I/R groups; (**f**) Ex vivo fluorescence images of kidneys from Sham, I/R + P, and I/R groups; (**g**) Quantitative analysis of enhanced fluorescence ratios and corresponding protein analysis of kidney samples. Copyright 2018, Elsevier B.V.

**Figure 12:**
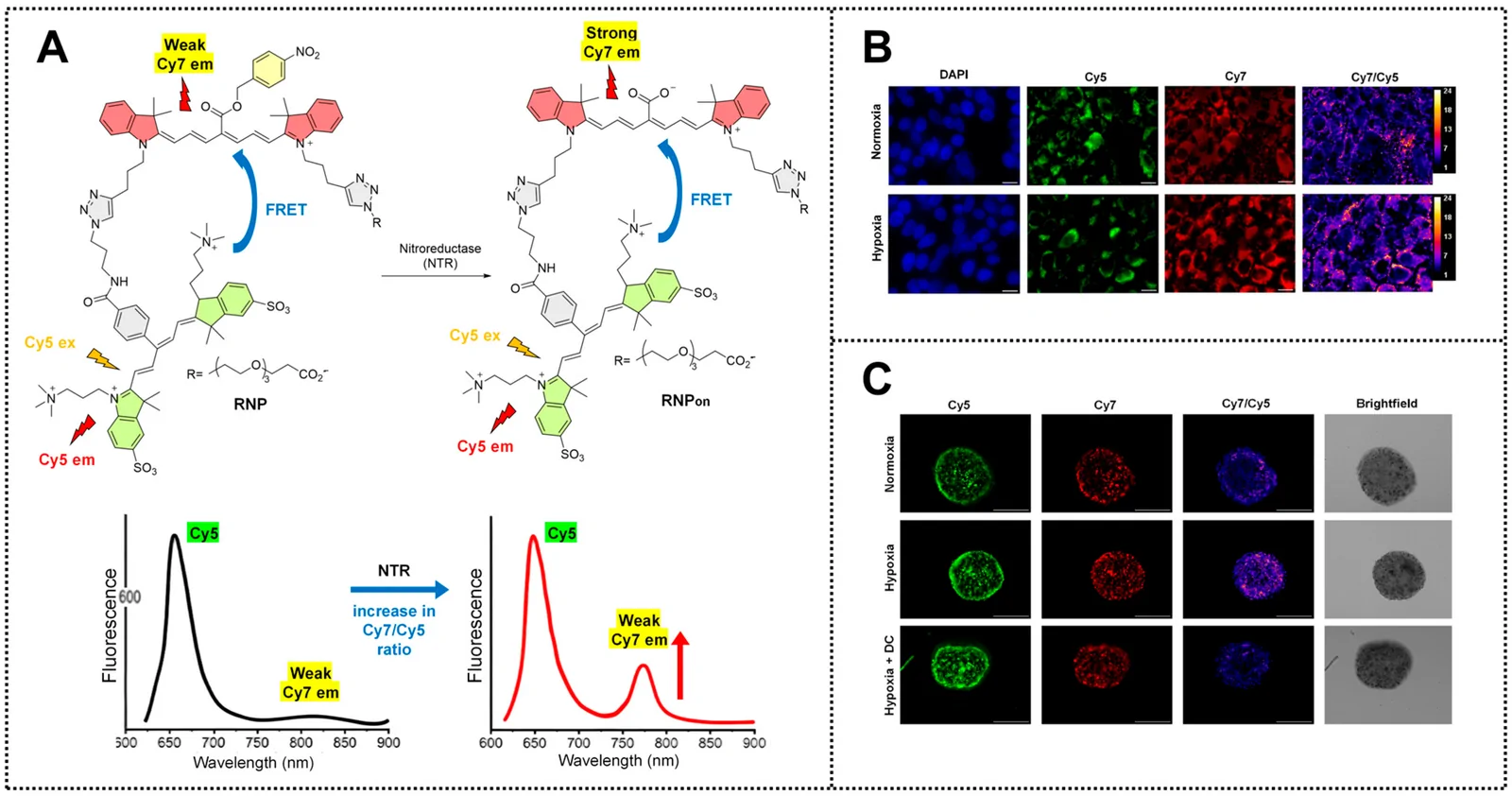
Hypoxia-associated imaging with Probe 8. (**A**) Molecular design and NTR-triggered response. (**B**) Fluorescence images of A549 cells. (**C**) Fluorescence images of three-dimensional HT-29 tumor spheroids. Copyright 2020, Royal Society of Chemistry.

**Figure 13:**
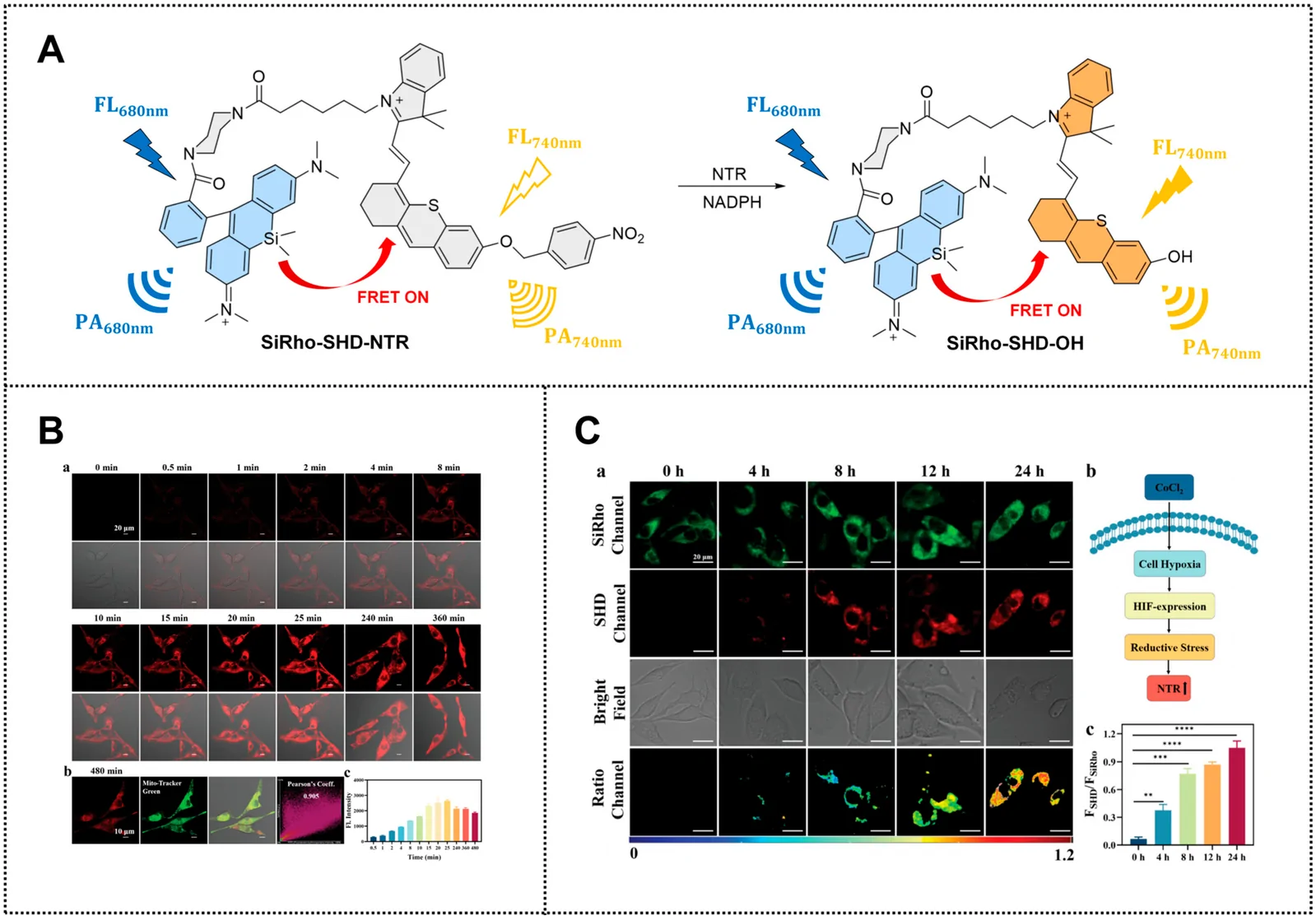
Hypoxia-associated imaging with Probe 9. (**A**) NTR-triggered reaction in the presence of NADH or NADPH. (**B**) Fluorescence imaging of pancreatic cancer cells with 5 μM Probe 9. (**a**) Short-term and long-term imaging of Pan02 cells using Probe 9. Scale bars: 20 μm; (**b**) Confocal imaging and mitochondrial colocalization imaging of Pan02 cells incubated with Probe 9 for 480 min. Scale bars: 10 μm; (**c**) Quantification of fluorescence intensity in confocal images of Pan02 cells from inset (**a**,**b**). (**C**) Fluorescence imaging of CoCl2 induced hypoxia in Pan02 cells: (**a**) Confocal imaging of Pan02 cells incubated with Probe 9. Scale bars: 20 μm; (**b**) Schematic diagram of the mechanism of CoCl2-induced cellular hypoxia; (**c**) Quantification of the fluorescence intensity ratio of the SHD Channel to the SiRho Channel in inset (**a**). Independent two-tailed *t*-tests indicate the following levels of significance: ** *p* < 0.01, *** *p* < 0.001, and **** *p* < 0.0001. Copyright 2024 Wiley-VCH GmbH.

**Figure 14:**
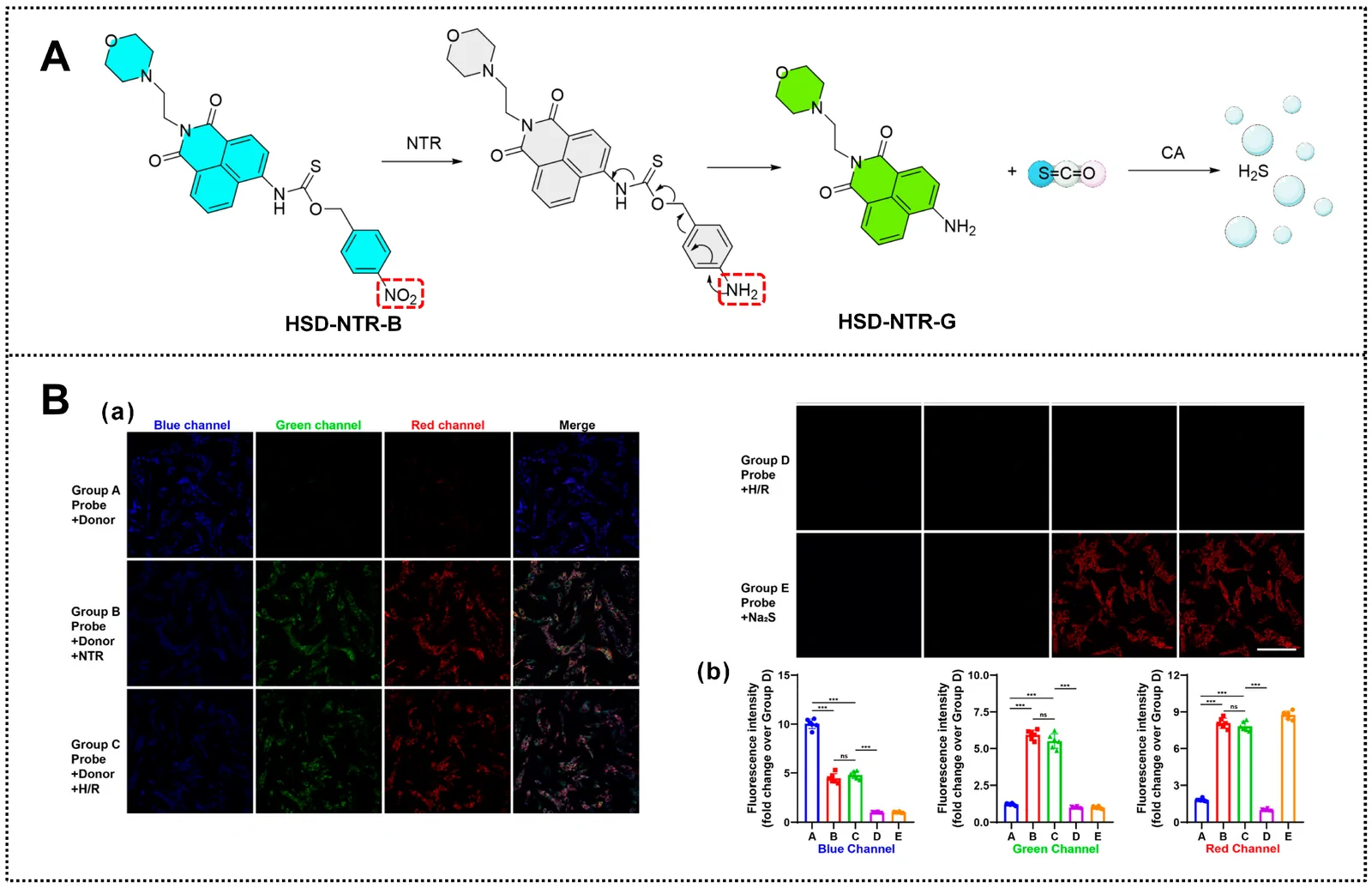
Hypoxia-associated imaging with Probe 15, an NTR-responsive fluorescent H_2_S donor. (**A**) Proposed activation and H_2_S-release mechanism. (**B**) Fluorescence imaging of H_2_S release in vitro: (**a**) Confocal fluorescence images of H9c2 cells after different treatments; (**b**) Quantitative analysis of fluorescence intensity changes in the blue, green, and red channels for Groups A–E. ns *p* ≥ 0.05, and *** *p* < 0.001. Copyright 2025, American Chemical Society. Copyright 2025, American Chemical Society.

**Figure 15:**
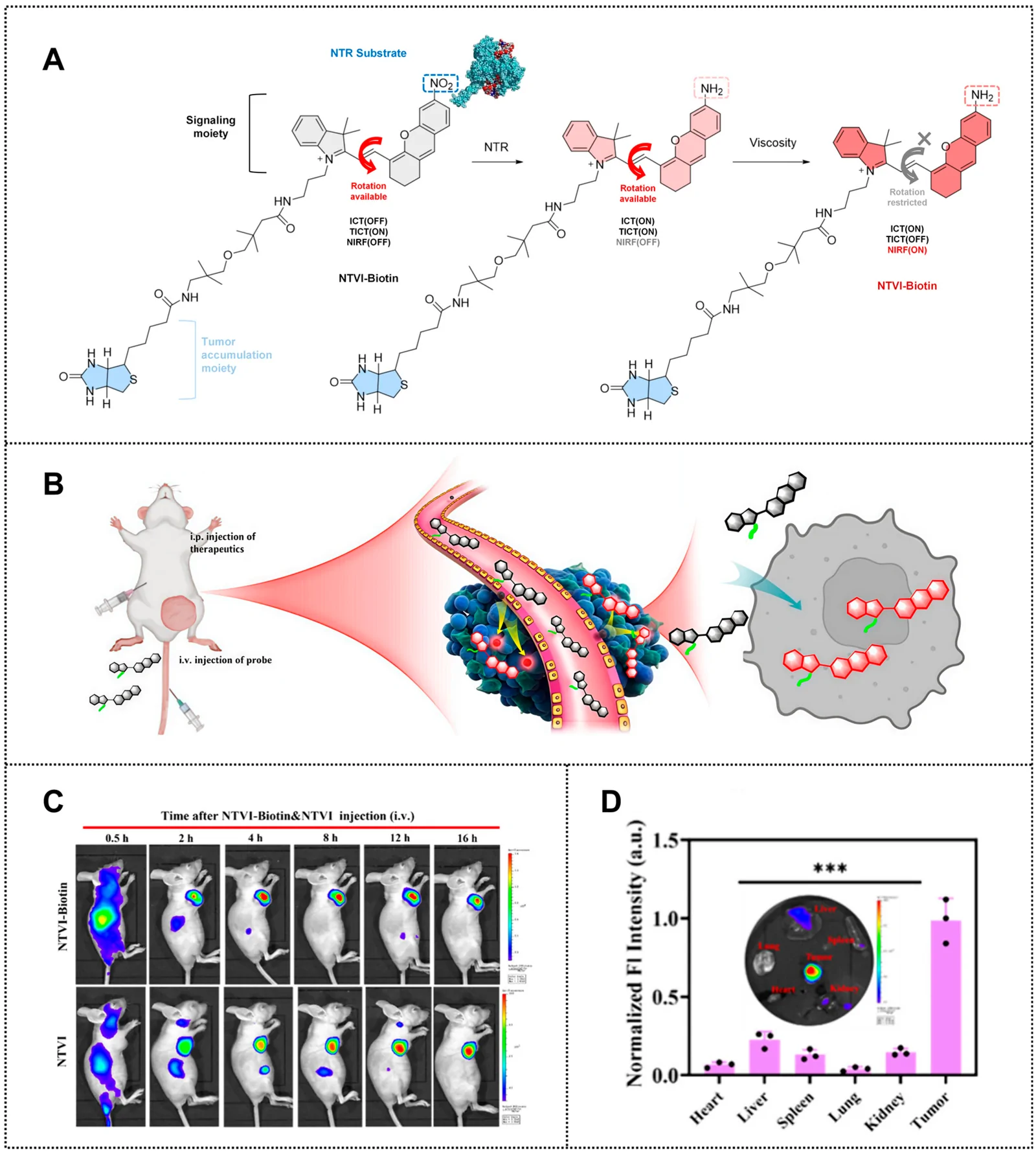
Hypoxia-associated imaging with Probe 17 (NTVI-Biotin). (**A**) Chemical structure and activation mechanism. (**B**) Targeting and cellular imaging workflow. (**C**) In vivo application and biodistribution. (**D**) Normalized ex vivo fluorescence intensity of tumor and major organs. *** *p* < 0.001. Copyright 2024, American Chemical Society.

**Figure 16:**
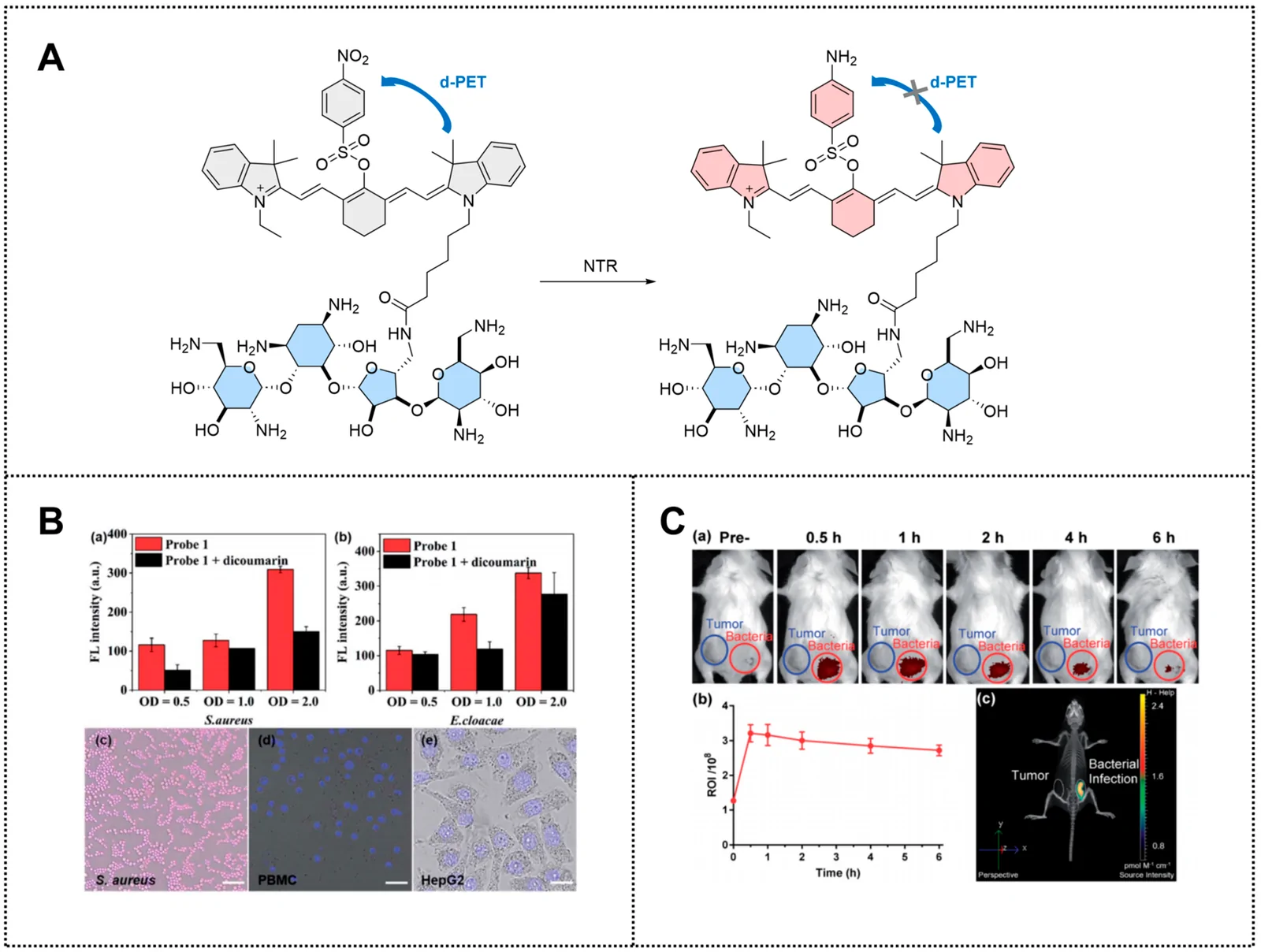
NTR-responsive bacterial imaging and infection-tumor discrimination using Probe 21 (Probe 1 in the source article). (**A**) Bacterial reduction of the nitro group and fluorescence activation. (**B**) Quantification analysis of the fluorescence intensity of probe 16 (10 mM) after incubation with (**a**) *S. aureus* and (**b**) *E. cloacae* in the presence (black bars) or absence (red bars) of the NTR inhibitor dicoumarin (1 mM); Merged confocal fluorescence microscopy images of (**c**) *S. aureus*, (**d**) PBMC and (**e**) HepG2 incubated with probe 16 (10 mM). Grey: bright-field; blue signals: Hoechst 33342 stain; red signals: probe 1, scale bar = 8 mm (**c**), 25 mm (**d**,**e**). (**C**) In vivo imaging for discrimination between bacterial infection and tumor: (**a**) Whole-body fluorescence images acquired after intravenous injection of Probe 16 into mice; (**b**) Time-dependent region-of-interest (ROI) ratio analysis of bacterial infection versus tumor signal; (**c**) Three-dimensional fluorescence light imaging tomography combined with micro-CT. Copyright 2020, Royal Society of Chemistry.

**Figure 17:**
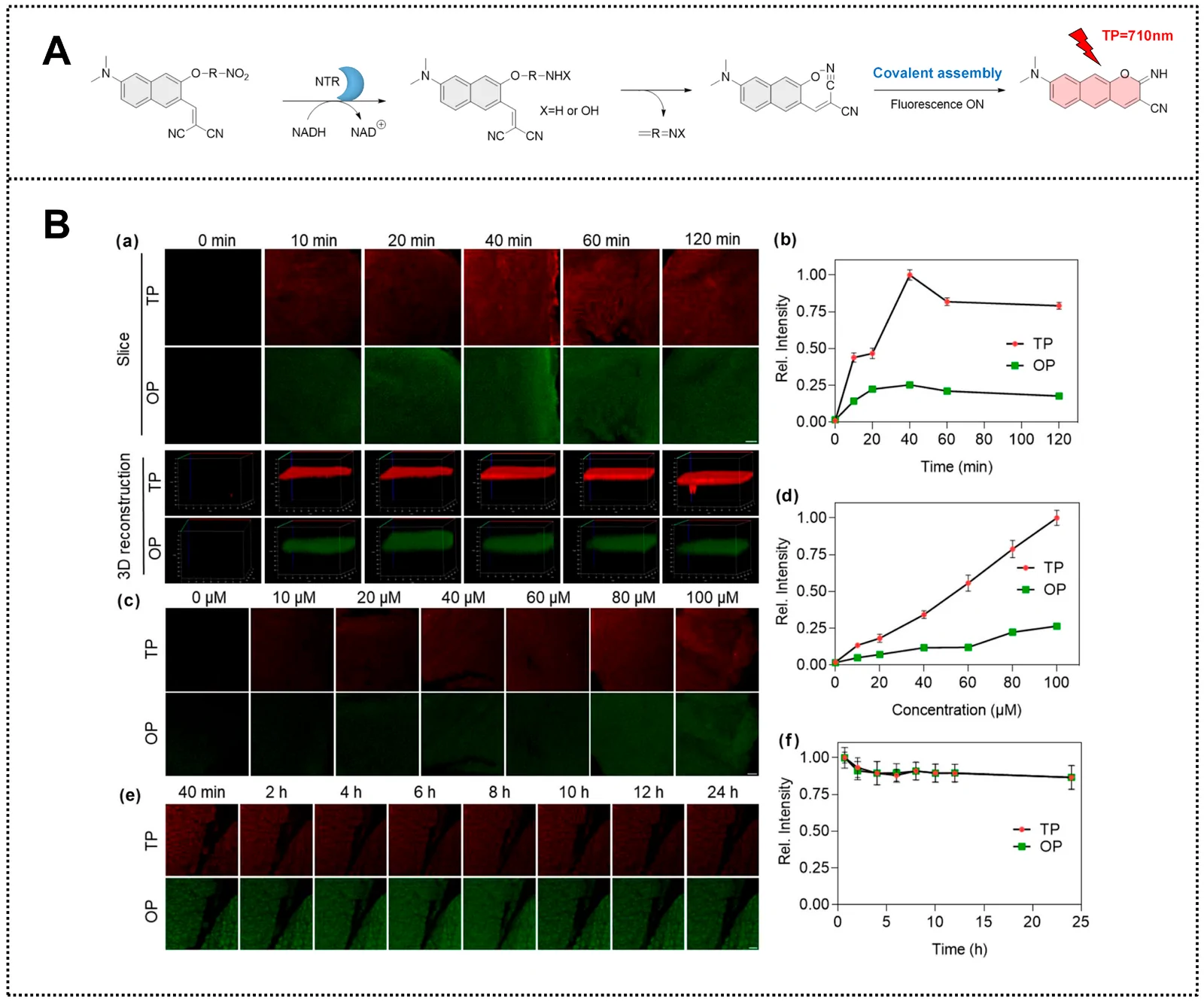
Hypoxia-associated imaging with Probe 24. (**A**) Principle of NTR-activated covalent assembly. (**B**) Two-photon (TP) and one-photon (OP) imaging of HepG2 tumor tissues treated with Probe 24: (**a**) Time-dependent TP and OP fluorescence images of mouse HepG2 tumor slices incubated with Probe 24, together with the corresponding three-dimensional reconstructions; (**b**) Quantitative analysis of the relative fluorescence intensity in the TP and OP channels corresponding to panel a; (**c**) TP and OP fluorescence images of mouse HepG2 tumor slices incubated with increasing concentrations of Probe 24 (0–100 μM); (**d**) Quantitative analysis of the relative fluorescence intensity in the TP and OP channels corresponding to panel c; (**e**) TP and OP fluorescence images of tumor slices; (**f**) Quantitative analysis of the relative fluorescence intensity in the TP and OP channels corresponding to the stability study in panel e. Copyright 2024, Elsevier B.V.

**Figure 18:**
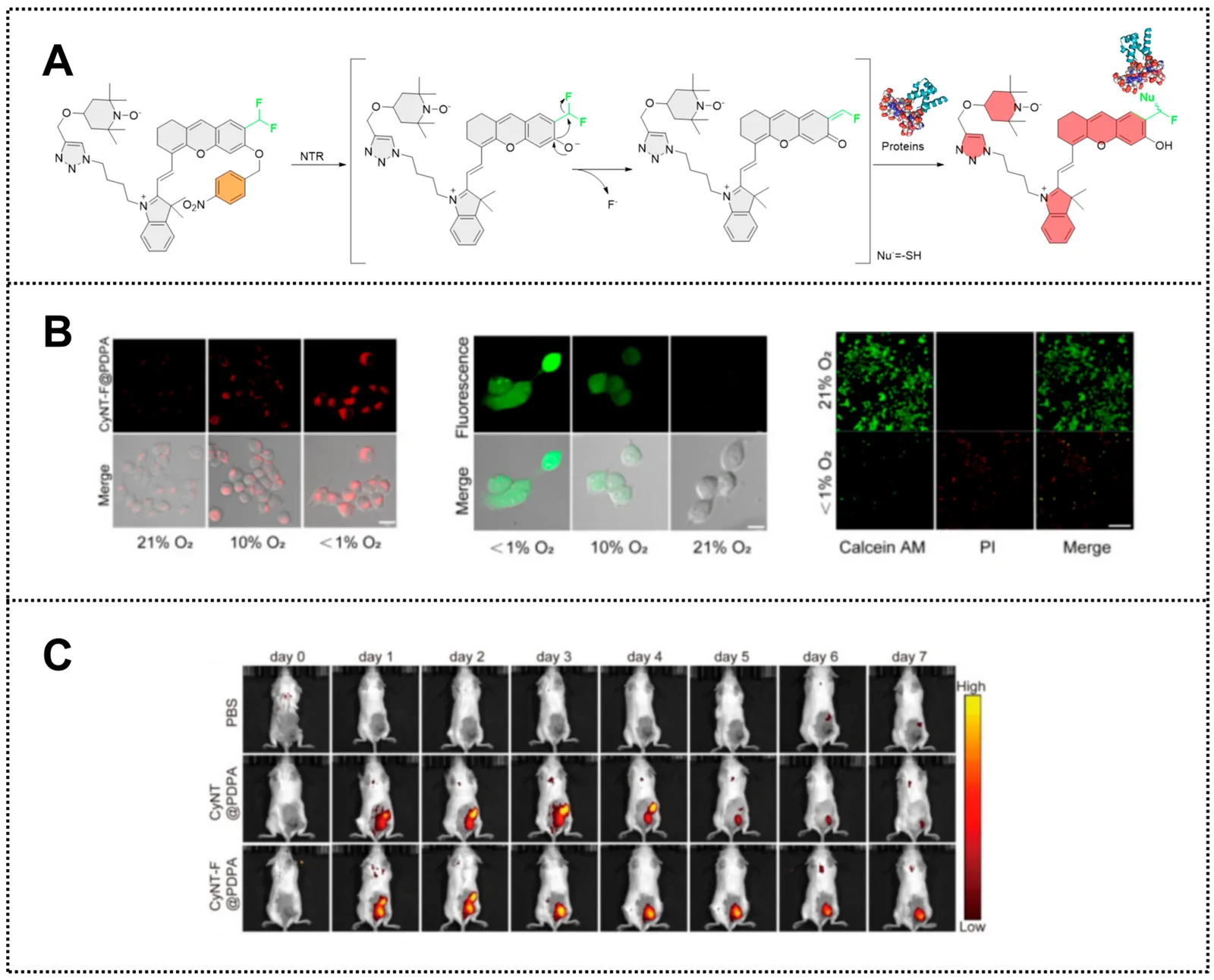
Hypoxia-associated imaging with Probe 26. (**A**) NTR-triggered response and protein-labeling mechanism. (**B**) Cellular uptake, ROS generation, and viability under different oxygen conditions. (**C**) Fluorescence imaging and retention in tumor models. Copyright 2024, American Chemical Society.

**Figure 19:**
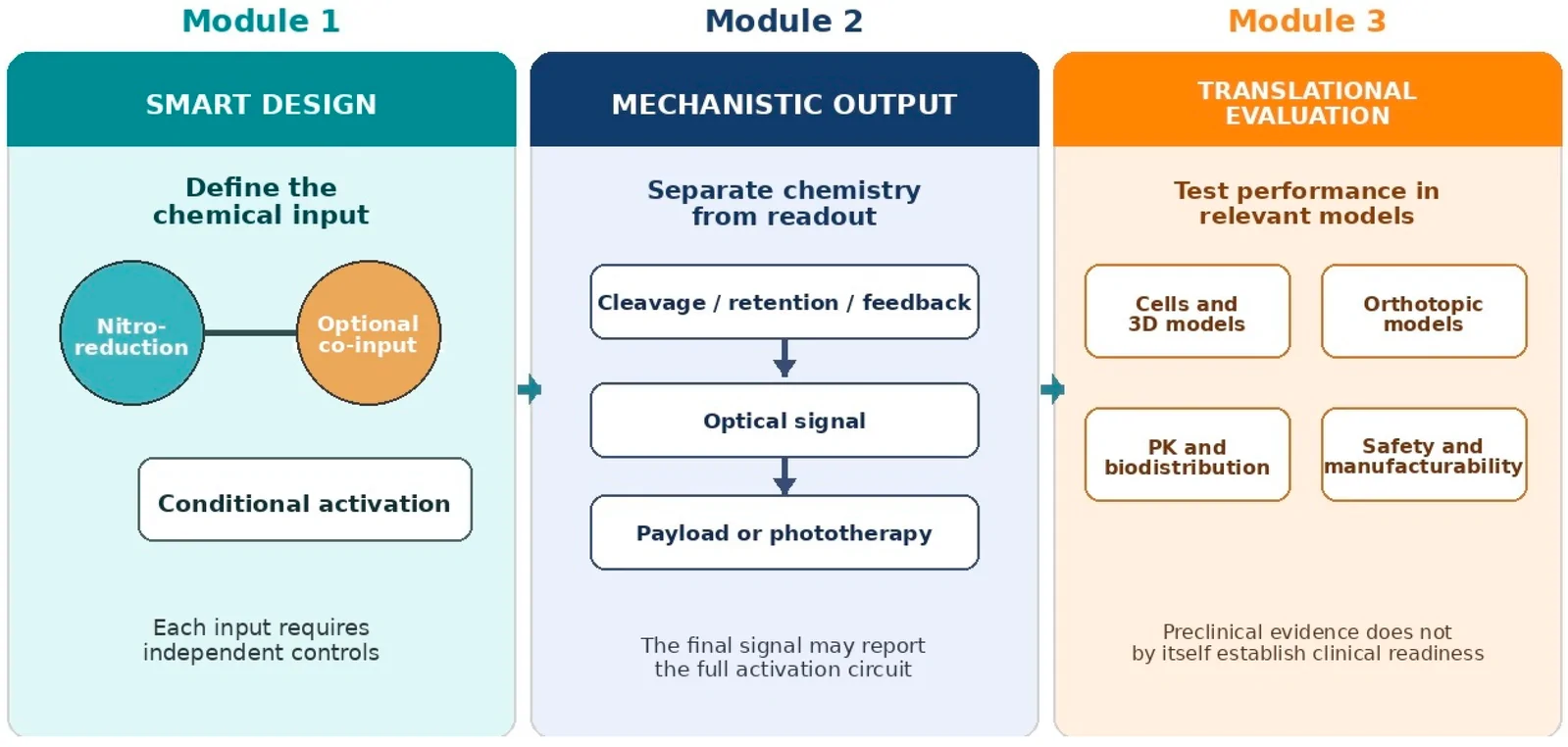
Conceptual NTR-activatable platform integrating nitroreduction-dependent imaging, conditional gating, and localized therapeutic activation. The final stage represents preclinical evaluation rather than established clinical translation. Author-created schematic.

**Figure 20:**
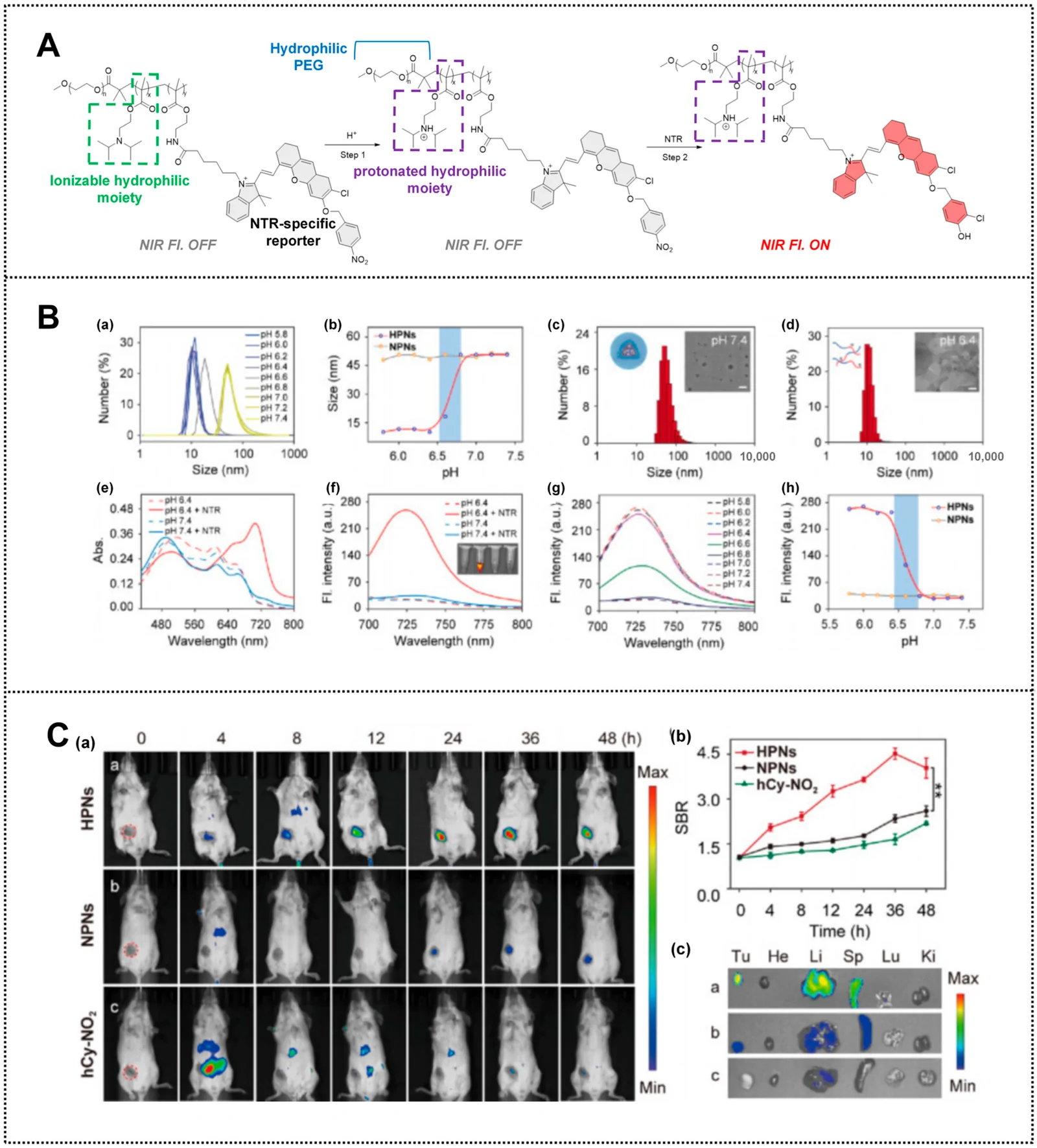
NTR-responsive imaging with Probe 11 nanomicelles under hypoxic conditions. (**A**) Sequential activation by acidic pH and NTR. (**B**) Cellular fluorescence imaging under control and activating conditions. (**C**) In vivo fluorescence imaging of tumors. (**B**) Physicochemical characterization and in vitro activation behavior of HPNs and control NPNs. (**a**) Size distributions of HPNs at different pH values, showing pH-responsive size transition. (**b**) pH-dependent particle size changes of HPNs and NPNs. (**c**,**d**) Representative size distributions/TEM images of the nanomicelles at pH 7.4 and 5.4, respectively. (**e**) Absorption spectra of HPNs under different pH conditions with or without NTR. (**f**) Fluorescence spectra of HPNs under pH 6.4 or 7.4 in the absence and presence of NTR, showing efficient fluorescence activation only under cascade conditions. (**g**) Fluorescence response of HPNs over a range of pH values. (**h**) pH-dependent fluorescence intensity profiles of HPNs and NPNs, confirming selective cascade activation of HPNs. (**C**) In vivo fluorescence imaging of the programmable stimulus activation in a 4T1 subcutaneous xenograft model. (**a**) Time-dependent whole-body fluorescence images of mice after administration of HPNs, NPNs, or the small-molecule control hCy-NO2 at 0, 4, 8, 12, 24, 36, and 48 h; red circles indicate tumor regions. (**b**) Quantification of tumor-to-background signal ratio (SBR) over time for the three groups. (**c**) Ex vivo fluorescence imaging of tumors and major organs harvested at 48 h, including tumor (Tu), heart (He), liver (Li), spleen (Sp), lung (Lu), and kidney (Ki), showing superior tumor-selective activation and retention of HPNs. Copyright 2024, American Chemical Society. Copyright 2024, American Chemical Society.

**Figure 21:**
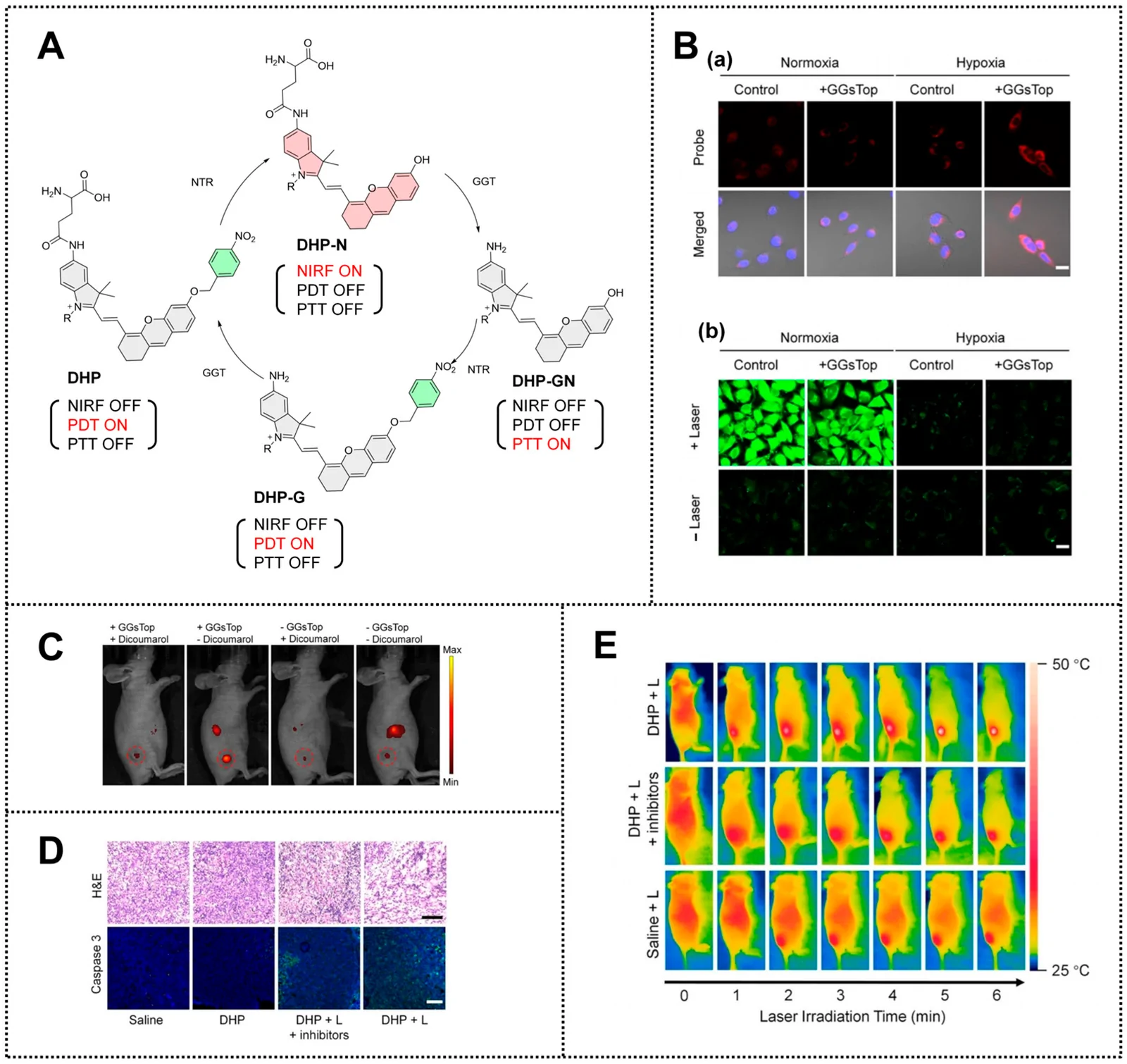
DHP dual-locked theranostic system. (**A**) Molecular mechanism for tumor imaging and autoregulated photodynamic therapy (PDT) and photothermal therapy (PTT) in the presence of gamma-glutamyl transferase (GGT) and NTR. (**B**) Cellular fluorescence imaging under the indicated conditions: (**a**) Representative confocal fluorescence images after incubation with DHP. (**b**) Intracellular ROS generation assessed by DCF fluorescence in cells. (**C**) In vivo tumor imaging and treatment. (**D**) Histological evaluation of tumor sections after treatment. (**E**) Infrared thermal imaging of tumor-bearing mice. Copyright 2022, Wiley-VCH GmbH.

**Figure 22:**
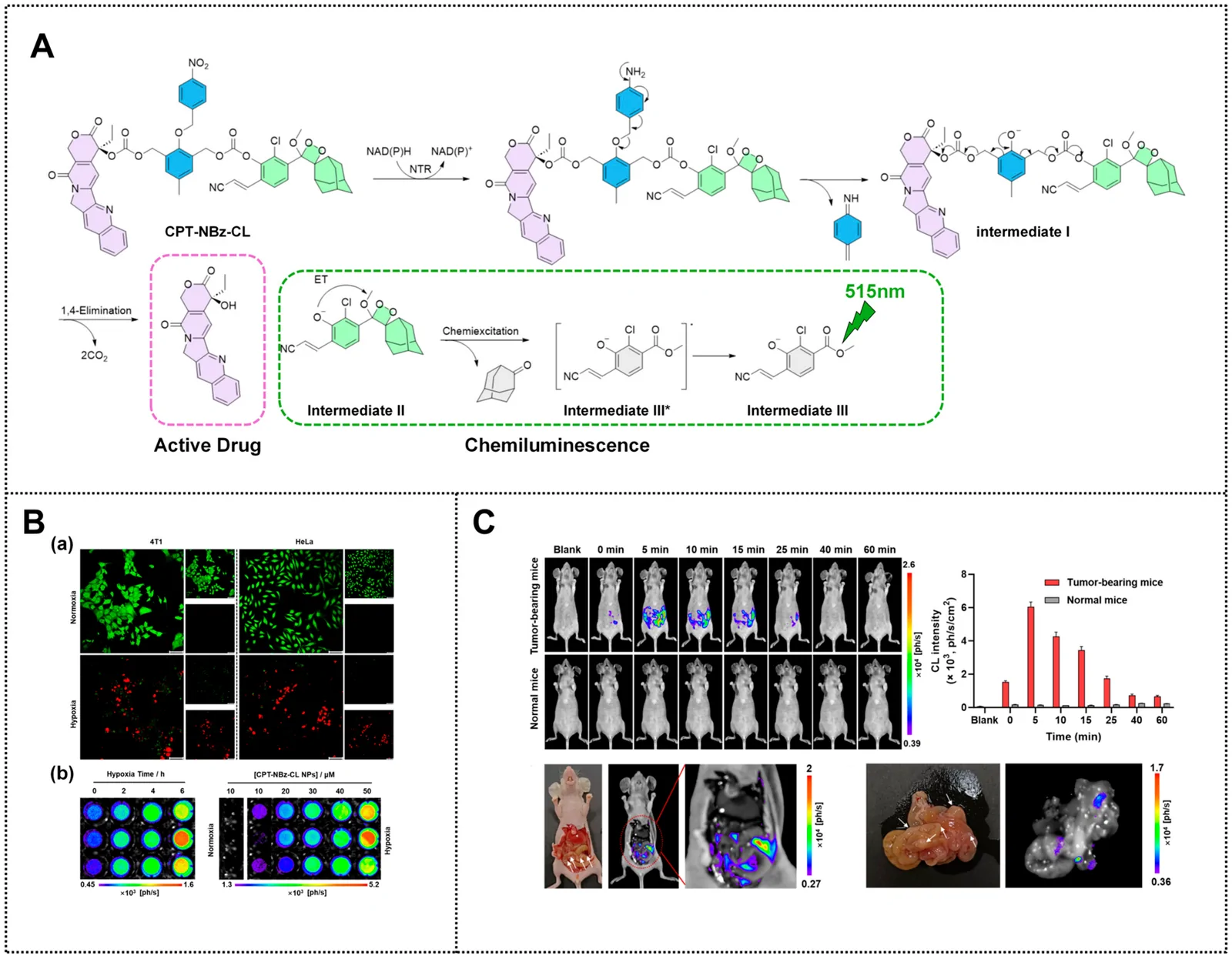
NTR-responsive chemiluminescent prodrug activation with Probe 27. (**A**) Proposed activation mechanism. (**B**) Mechanism of action and in vitro chemiluminescence imaging of Probe 27 nanoparticles: (**a**) Fluorescence/live-dead images of 4T1 and HeLa cells; (**b**) In vitro chemiluminescence images of Probe 27. (**C**) Chemiluminescence imaging of 4T1 tumor-bearing mice. Copyright 2025, American Chemical Society.

**Figure 23:**
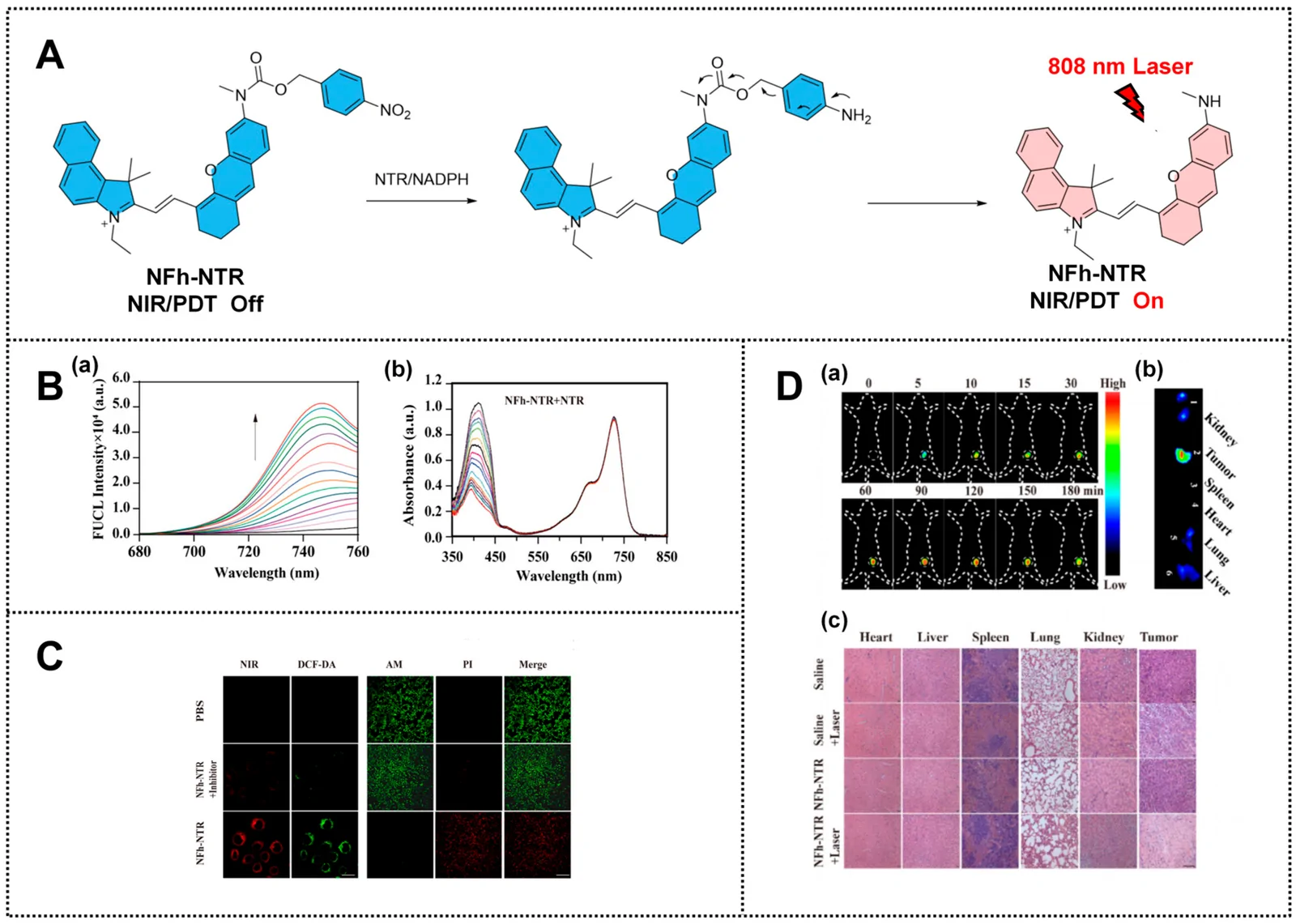
NTR-responsive activatable photodynamic therapy with NFh-NTR. (**A**) Activatable design and proposed mechanism. (**B**) Spectroscopic characterization of NTR-triggered activation and singlet-oxygen generation. (**a**) Time-dependent frequency upconversion luminescence (FUCL) spectra of NFh-NTR. (**b**) Absorption spectral changes of NFh-NTR. (**C**) Cellular imaging and in vitro photodynamic evaluation. (**D**) In vivo imaging, biodistribution, and histological evaluation. (**a**) Time-dependent in vivo NIR fluorescence imaging of tumor-bearing mice. (**b**) Ex vivo fluorescence imaging of major organs and tumor. (**c**) H&E staining of heart, liver, spleen, lung, kidney, and tumor sections from different treatment groups. Copyright 2024, American Chemical Society.

**Figure 24:**
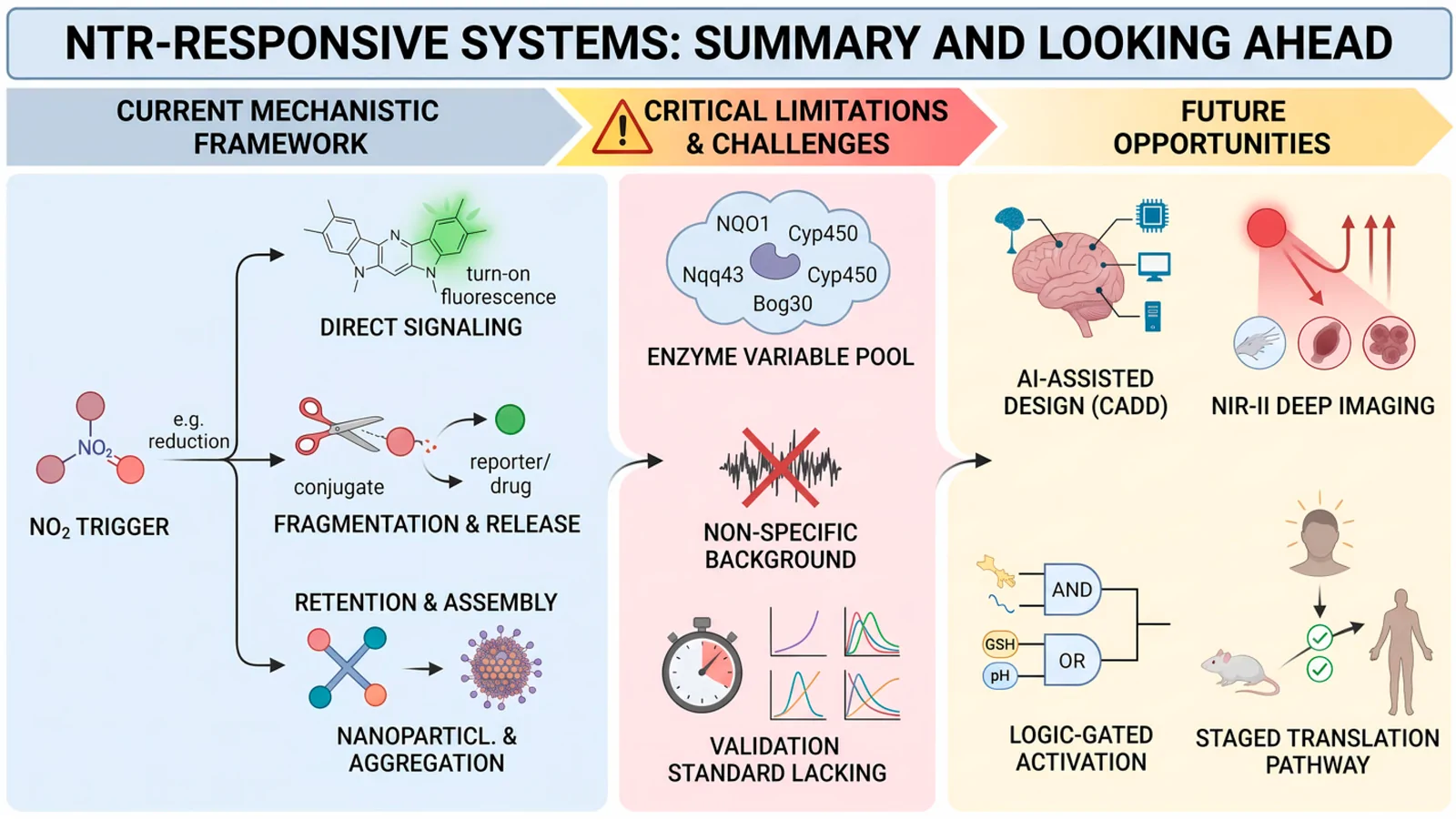
Summary of the mechanistic framework, key limitations, and future directions of NTR-responsive systems, highlighting major reaction architectures, validation challenges, and emerging strategies including AI/CADD, NIR-II imaging, logic-gated activation, and staged translation.

**Table 1:** Direct comparison of recent reviews on NTR-responsive systems and the present review [1,2,6,7].

Review	Scope and Biological Systems	Classification and Imaging Modalities	Therapeutic Applications	Time Coverage and Distinctive Emphasis
Tran et al. (2025) [1]	NTR-activated probes for hypoxia monitoring; principally mammalian tumor models.	Probe design and fluorescence-based hypoxia monitoring.	Not the principal organizing focus.	2025; imaging-focused review.
Shang et al. (2024) [2]	NTR-activable agents for tumor theranostics.	Agents and applications; fluorescence and tumor imaging.	Tumor theranostics, conditional drug delivery, and phototherapy.	2024; tumor-theranostic focus.
Verma et al. (2024) [6]	Roles of NTRs in bacterial, animal, and plant biosystems.	Biological roles and fluorescent-probe detection.	Not the central focus.	2024; broad biosystems survey.
Ji et al. (2026) [7]	NTR-activated fluorescent probes from molecular design to disease diagnosis.	Molecular design and fluorescence-based disease diagnosis.	Not the central focus.	2026; diagnosis-focused review.
Present review	Defined bacterial NTRs, engineered NTR systems, and endogenous mammalian nitro-reducing activity.	Catalytic source → molecular modules → reaction architecture → signal transduction → biological application; fluorescence, PA, chemiluminescence, and ^19^F NMR.	Conditional payload release, PDT, PTT, and multimodal therapy.	Includes publications through 2026; separates chemical event, signal transduction, and biological inference.

**Table 2:** Biological contexts, plausible catalytic sources, and the limits of interpretation for nitroreductase-responsive systems.

Biological Setting	Catalytic Source and Reducing Equivalents	Relationship to Oxygen	Claim Supported	Claim Not Supported
Purified bacterial Type I NTR	Defined FMN-dependent enzyme, commonly NfsA/NfsB; NADH or NADPH [3,4]	Productive two-electron turnover can occur in air; magnitude remains enzyme- and substrate-dependent.	Substrate turnover, cofactor dependence, kinetic mechanism, and product identity.	Identity of an endogenous mammalian catalyst or quantitative tissue hypoxia.
Endogenous mammalian cells or tissues	Substrate-dependent mixture that may include NQO1, cytochrome P450 oxidoreductase, and other flavoprotein reductases [1,2]	Oxygen competes with one-electron pathways; the net effect varies with probe structure, compartment, and metabolism.	Operational nitro-reducing activity under the stated biological and oxygen conditions.	A universal “tumor NTR,” exclusive NQO1 attribution, or direct oxygen partial pressure.
Native bacterial detection	Species- and growth-state-dependent microbial reductase complement coupled to native NAD(P)H metabolism [17,18,20,21,22,23,24]	Depends on organism, growth phase, burden, and local oxygenation.	Organism-associated nitroreduction and lesion-level diagnostic contrast.	A universal bacterial enzyme mechanism or direct comparability with mammalian tumors.
Engineered NTR expression or enzyme–prodrug therapy	Introduced bacterial NTR transgene with a defined substrate or prodrug [5,17,18]	Determined by the selected enzyme–substrate pair and expression context.	Causal enzyme-controlled activation, ablation, or prodrug conversion.	Evidence that endogenous mammalian probes report the same enzyme system.

**Table 3:** Analytical characteristics, model context, and interpretive boundaries of representative PET- and ICT-controlled fragmentation systems.

System	System’s Structure	Solvent	λ_ex_ (nm)	λ_em_ (nm)	Response Time (min)	Detection Limit	Model/Reported Validation/Interpretive Boundary
1 [45]	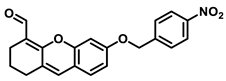	PBS buffer (pH 7.4, 10 mM)	420	540	10	5.651 ng/mL	4T1 cells and tumor-bearing mice; inhibitor control reported. Signal also depends on delivery and clearance.
2 [46]	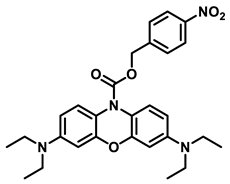	PBS buffer (pH 7.4, 10 mM)	635	683	10	0.09 μg/mL	Thyroid-cancer imaging/surgical guidance; endogenous activating enzyme was not uniquely resolved.
3 [26]	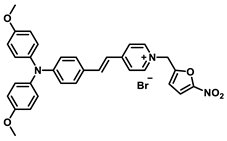	1× PBS (pH 7.4) or DMSO/PBS;	400	540	30	-	Cellular and 4T1 PDT models; aggregation and oxygen jointly affect fluorescence and ROS.
4 [27]	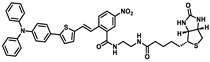	DMSO/toluene	458	668	-	-	Hypoxic-tumor AIE imaging; incomplete analytical reporting limits quantitative comparison.
5 [28]	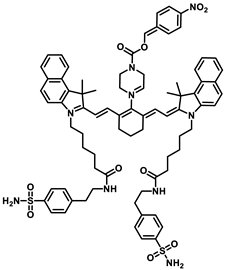	10% DMSO in PBS + 500 μM NADH	725	825	14	0.0161 μg/mL	Orthotopic lung cancer/metastasis; contrast depends on receptor expression and nitroreduction.
6 [29]	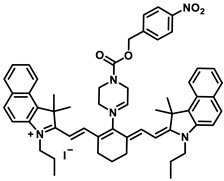	PBS (pH 7.4) + 1% DMSO	630	785	8	0.0242 μg/mL	Cells and tumor-bearing mice; oxygen-dependent NIR imaging. Product and enzyme attribution remain incomplete.

**Table 4:** Analytical characteristics, model context, and interpretive boundaries of representative FRET, ratiometric, and therapy-coupled systems.

System	System’s Structure	Solvent	λ_e_x (nm)	λ_em_ (nm)	Response Time (min)	Detection Limit	Model/Reported Validation/Interpretive Boundary
8 [13]	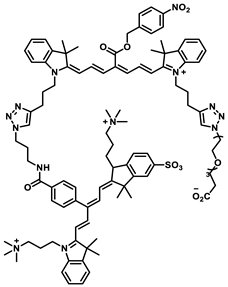	1× PBS (pH7.4)	620 (Cy5); 720 (Cy7)	660 (Cy5); 780 (Cy7)	30	0.3 μg/mL	A549 cells, HT-29 spheroids, and U87 models; ratiometric response. Cross-talk and conversion calibration remain necessary.
9 [38]	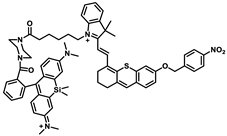	PBS/DMSO = 9/1 (pH7.4)	640 (SiRho) 740 (SHD)	680 (SiRho) 760 (SHD)	5–10	50 ng/mL	Pancreatic-cancer fluorescence/PA imaging; each modality requires independent calibration.
10	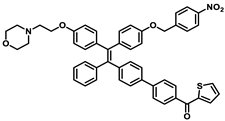	PBS/DMSO (pH7.4)	380	530	30	53.6 ng/mL	Lysosomal imaging and PDT in 4T1 models; probe-induced organelle perturbation should be excluded.
11 [48]	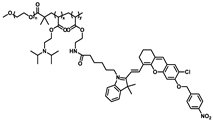	PBS (pH 7.4 or 6.4)	640	725	20	24 ng/mL	Orthotopic breast cancer/lung metastases; pH/NTR gating requires complete single-input controls.

**Table 5:** Analytical characteristics, model context, and interpretive boundaries of representative self-immolative release systems.

System	System’s Structure	Solvent	λ_ex_ (nm)	λ_em_ (nm)	Response Time (min) Time (M in)	Detection Limit	Model/Reported Validation/Interpretive Boundary
12 [30]	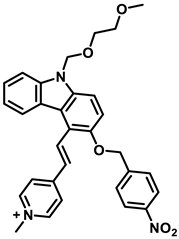	PBS + NADH	488	535	30	0.0138 μg/mL	HeLa mitophagy model; causal interpretation requires exclusion of probe-induced mitochondrial perturbation.
13 [31]	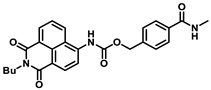	PBS + NADH	405	540	-	-	Mitochondrial amine release; release yield and target-independent activation require quantification.
14 [32]	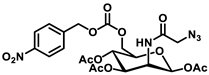	-	-	-	-	-	Transgenic zebrafish/covalent labeling; signal also depends on accessible labeling targets.
15 [33]	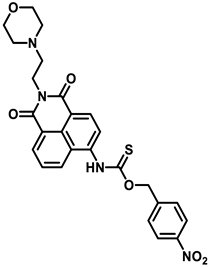	PBS + NADH + CA	410	475	60	-	Myocardial ischemia–reperfusion; H_2_S release and nonspecific activation require direct testing.

**Table 6:** Analytical characteristics, model context, and interpretive boundaries of representative direct-reduction systems.

System	System’s Structure	Solvent	λ_ex_ (nm)	λ_em_ (nm)	Response Time (min)	Detection Limit	Model/Reported Validation/Interpretive Boundary
16 [34]	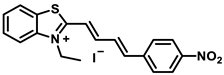	PBS (pH 7.4) + 500 μM NADH	515	664	420	0.0368 μg/mL	HeLa, SH-SY5Y, and A549 cells; viscosity sensitivity and long incubation limit enzyme-rate interpretation.
17 [39]	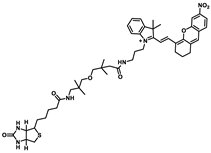	PBS with glycerol/ethanol	630	710	50	170 ng/mL	Targeted in vivo deployment; contrast combines ligand expression, delivery, and nitroreduction.
18 [40]	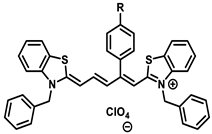	DCM	685	690	120	-	Mitochondrial hypoxia imaging; each gate input requires independent validation.
19 [41]	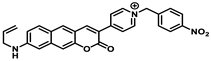	PBS (pH 7.4) + NADH	475	655	30	25 ng/mL	Cancer/normal and senescent cells; binding equilibrium must be separated from enzyme turnover.
20 [42]	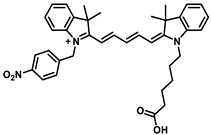	0.05 M Tris (pH 7.4) + 1.5% DMSO	620	657	60	36.9 ng/mL	Live *S. aureus*/MRSA; selectivity depends on bacterial load, growth phase, oxygen, and host background.
21 [24]	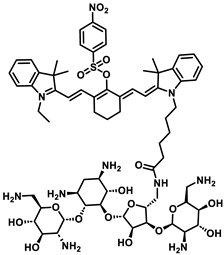	Tris-buffered saline (pH 7.4) + 1% DMSO	750	809	1	0.67 ng/mL	Infection/cancer discrimination in mice; signal remains burden- and delivery-dependent.
22 [35]	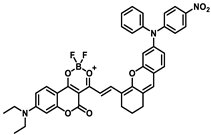	PBS (pH 7.4) + 5% DMSO	680	791	-	0.048 μg/mL	Orthotopic liver cancer/navigation; fluorescence and aggregation require tissue-specific calibration.
23 [36]	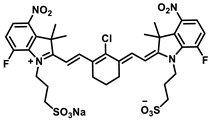	PBS + NADH	745	773	60	-	Orthotopic lung cancer; fluorescence and ^19^F NMR require independent calibration.

**Table 7:** Analytical characteristics, model context, and interpretive boundaries of representative retention, covalent-capture, and chemiluminescent systems.

System	System’s Structure	Solvent	λ_ex_ (nm)	λ_em_ (nm)	Response Time (min)	Detection Limit	Model/Reported Validation/Interpretive Boundary
24 [37]	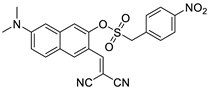	PBS (0.01 M, pH 7.4) + 5% DMSO + NADH	485	540	30	59 ng/mL	Cancer cells/human colorectal tissues; covalent assembly prevents instantaneous enzyme interpretation.
25 [51]	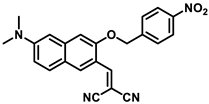	PBS (10 mM, pH 7.4) + 5% DMSO	490	595	60	0.142 ng/mL	Cells, zebrafish, mice, and human tissues; two-photon excitation improves sectioning, not universal depth.
26 [44]	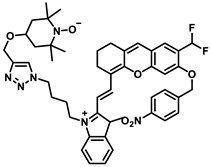	PBS (pH 7.4)	640	720	10	-	Hypoxic-tumor PDT/protein labeling; retention raises off-target binding and clearance questions.
27 [52]	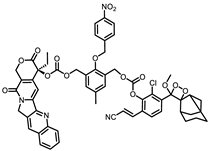	PBS (10 mM, pH 7.4) + 1% DMSO	-	521	22	0.66 μg/mL	4T1 peritoneal metastasis; chemiluminescence should be calibrated against drug release and dose.

**Table 8:** Cross-architecture decision matrix for NTR-responsive systems.

Architecture	Best Suited for	Principal Trade-Off	Essential Controls
Direct electronic reconfiguration	Rapid switching and compact reporters	Environmental sensitivity and limited payload modularity	Product confirmation; polarity/viscosity controls; concentration calibration
Reduction-triggered fragmentation	Low background and reporter or payload release	Multistep kinetics, incomplete release, and product diffusion	Conversion and release yield; by-product mapping; serum and dark stability
Retention, capture, or assembly	Spatial persistence, lesion mapping, and prolonged local therapy	Cumulative signal, target-abundance dependence, and clearance	Target/assembly characterization; washout or reversibility; biodistribution and histology

## Data Availability

No new data were created or analyzed in this study. Data sharing is not applicable to this article.
